# TargetSearch - a Bioconductor package for the efficient preprocessing of GC-MS metabolite profiling data

**DOI:** 10.1186/1471-2105-10-428

**Published:** 2009-12-16

**Authors:** Álvaro Cuadros-Inostroza, Camila Caldana, Henning Redestig, Miyako Kusano, Jan Lisec, Hugo Peña-Cortés, Lothar Willmitzer, Matthew A Hannah

**Affiliations:** 1 Max Planck Institute of Molecular Plant Physiology, Am Mühlenberg 1, D-14476 Potsdam-Golm, Germany; 2 Centro de Biotecnología, Universidad Técnica Federico Santa María, General Bari 699 Valparaíso, Chile; 3 RIKEN Plant Science Center, Tsurumi-ku, Suehiro-cho, 1-7-22 Yokohama, Kanagawa, 230-0045, Japan; 4 Bayer BioScience N.V., Technologiepark 38, B-9052, Gent, Belgium

## Abstract

**Background:**

Metabolite profiling, the simultaneous quantification of multiple metabolites in an experiment, is becoming increasingly popular, particularly with the rise of systems-level biology. The workhorse in this field is gas-chromatography hyphenated with mass spectrometry (GC-MS). The high-throughput of this technology coupled with a demand for large experiments has led to data pre-processing, i.e. the quantification of metabolites across samples, becoming a major bottleneck. Existing software has several limitations, including restricted maximum sample size, systematic errors and low flexibility. However, the biggest limitation is that the resulting data usually require extensive hand-curation, which is subjective and can typically take several days to weeks.

**Results:**

We introduce the *TargetSearch *package, an open source tool which is a flexible and accurate method for pre-processing even very large numbers of GC-MS samples within hours. We developed a novel strategy to iteratively correct and update retention time indices for searching and identifying metabolites. The package is written in the R programming language with computationally intensive functions written in C for speed and performance. The package includes a graphical user interface to allow easy use by those unfamiliar with R.

**Conclusions:**

*TargetSearch *allows fast and accurate data pre-processing for GC-MS experiments and overcomes the sample number limitations and manual curation requirements of existing software. We validate our method by carrying out an analysis against both a set of known chemical standard mixtures and of a biological experiment. In addition we demonstrate its capabilities and speed by comparing it with other GC-MS pre-processing tools. We believe this package will greatly ease current bottlenecks and facilitate the analysis of metabolic profiling data.

## Background

Metabolite profiling has become a powerful tool to address biological problems. It has been used in a wide range of applications, such as discovering the effects of herbicides in plants [1], drugs in medical fields [2,3], or molecular physiology and functional genomics [4-6].

GC-MS is by far the most widely applied analytical method as it provides an efficient way to quantify hundreds of metabolites in a single sample run [7-9]. In addition, robust extraction protocols exist and there is continued development of data evaluation methods. See [10] for a introduction to metabolomics using GC-MS.

The relatively low cost and high-throughput of GC-MS instruments has led to data pre-processing becoming a major bottleneck in metabolite profiling, particularly with recent increases in experiment size. The typical GC-MS pre-processing approach consists of peak detection and mass spectra deconvolution, retention time alignment across different samples and a normalisation step to remove systematic variation in the data. Finally, the peak information is collected and transformed into a data matrix for statistical analysis. Many software tools are available that can perform said steps, either using a non-targeted approach, i.e., all the extracted peaks are used as relevant information, or a metabolite-targeted approach, where detected peaks are searched in existing compound databases, matching their retention time and spectra. Examples of GC-MS pre-processing software solutions include AMDIS [11], Leco ChromaTOF [12], MetAlign [13], XCMS [14] and Tagfinder [15].

Although deconvolution is a well accepted method, biological complexity of samples, e.g., co-eluting compounds, causes errors in the deconvolution algorithm that result in partially deconvoluted or mixed mass spectra tags (MSTs). A widely accepted alternative to avoid this inconvenience is the extraction of peak apex intensities for selected masses [15]. This method has the advantage of being both less computationally intensive and less error susceptible. In spite of that, existing tools have other limitations such as restrictions to maximum sample number, long run-time and complicated peak identification. The latter typically requires manual inspection and curation that may take weeks to complete for an average experiment.

Our aim was to develop a flexible and automated tool that can pre-process data, identify and quantify metabolite levels for even very large numbers of samples in a targeted manner, whilst keeping processing time to a minimum. Our approach uses peak apex intensities to avoid deconvolution errors, a metabolite reference library based on selective masses and retention time indices [16], conversion and correction from retention time (RT) to retention time index (RI), RI updating and metabolite identification using multiple correlated masses [15,17].

Our software is implemented in the R language with computationally intensive functions programmed in C for speed and performance. The software is available as an R-package within the framework of the Bioconductor project [18].

## Implementation

**Figure 1:**
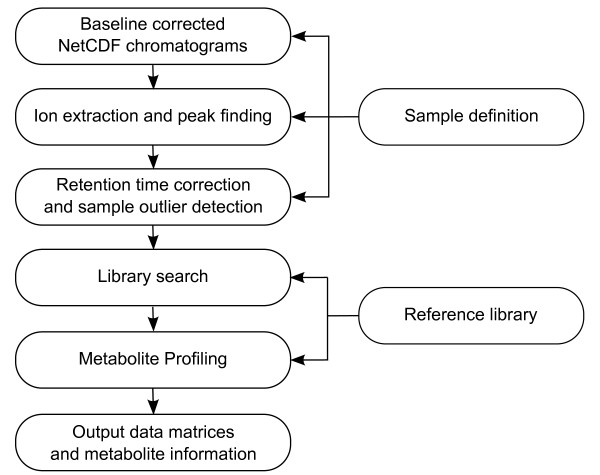
**TargetSearch flow chart**. *TargetSearch *pre-processing flow chart for the analysis of GC-MS data.

This section describes our GC-MS pre-processing approach (Figure 1). In short, a sample definition file and a reference library are required to identify the files to process and to provide the metabolite information to be searched for. Mass trace apices are extracted from every chromatogram and stored in text files. At the same time, RT is converted to RI. Selective masses from the reference library are searched in a three-step RI updating procedure. Every file, either chromatogram or peak list, is processed one at a time, allowing the analysis of a large number of samples. Also, since the most time consuming algorithms are written in C, the overall processing time, from raw chromatograms to a metabolite data matrix, is very short.

### User input

There are three types of input that the user must supply: chromatogram files, sample description and a reference library.

GC-MS chromatograms must be first exported to the widely accepted and platform-independent NetCDF file format. This is usually performed by using the platform-specific GC-MS software (e.g. Leco ChromaTOF). Baseline correction and smoothing could be performed before exporting the chromatograms, but as this is not always done, we implemented these steps so they can be performed by *TargetSearch*.

The "sample description" file contains a list of sample names, sample files and sample groups, which is used for the downstream analyses.

Finally, the "reference library" file contains the information on the MSTs that will be searched for in the chromatograms. Required information is the metabolite name, expected RI, selective masses, most abundant masses, spectra and RI deviation. The file format can be tab-delimited text containing said columns or the commonly used NIST MS Search Software text format [19]. We developed a novel iterative approach to RI assignment to take advantage of the fact that RI variation decreases in the following order: between experiment *>> *between samples *>> *between masses from the same metabolite. Thus, we use selective masses to identify the experiment RI from the library RI, following that use this to find the sample RI and finally use this to identify co-eluting masses for quantification. *TargetSearch *can be used with freely available public metabolite libraries, for example, in [16,20], with commercial libraries, for example, [21] or in-house developed or curated libraries.

### Peak identification and RI correction

Initially, *TargetSearch *performs baseline correction and identifies the local apex intensities from the mass traces in all chromatograms. The baseline correction algorithm is based on [22] and should be performed if the exported chromatograms were not baseline corrected by the vendor software. This is controlled by the user. After that, *TargetSearch *finds the retention time of the RI markers, internal standard compounds like alkanes or fatty acid methyl esters, and converts the retention time to RI using a linear interpolation [23]. The RI marker definition, time window and *m/z *values, have to be provided by the user in the same way as for other software [12,15,24].

In addition, a report is created to allow the rapid assessment of RI marker outliers. This is an essential step which can be cumbersome and time-consuming using other methods. The generated report contains a separate plot for each retention index marker in which sample number is plotted against the found retention time. Outliers are highlighted on a per-day basis using a 3*σ *cut-off (or other defined cut-off). Here, measurement days are defined in the "sample description" file or can be detected automatically using hierarchical clustering. This report allows a fast assessment of outliers allowing the user to quickly decide whether the sample should be removed from the data set or not. It is also possible to manually edit the found retention time, but this is in our experience rarely necessary.

Finally, *TargetSearch *creates a tab delimited file per chromatogram file which contains a peak list, retention times and converted RIs. For efficiency, it is possible to start subsequent steps from these saved files by selecting "Apex Data" instead of "NetCDF Data".

### Library search

Metabolites are identified in three steps. First, for every metabolite, selective masses are searched for in a given time window around the expected RI, returning the median RI of all selective masses. The expected RI of the respective metabolite is updated to that value. In the second step, *TargetSearch *searches the selected masses using the updated RI and a given time deviation, usually smaller than the one used in the first step. The intensities of the selected masses are normalised to the median of the day, and then used to extract other masses with correlated apex profiles. The masses for which the Pearson correlation coefficient is above a given threshold are taken as metabolite markers and their RIs are averaged on a per sample basis. This average RI is taken to represent the exact position where the metabolite elutes in the respective sample. Finally, using this exact RI and a much smaller RI window, *TargetSearch *searches again for the top masses and the apex intensities are returned.

### Metabolite profile

The aim of *TargetSearch *is to return a metabolite profile that is directly interpretable with little additional manual curation. This is achieved by using many correlated masses for accurate metabolite identification and quantification. *TargetSearch *details the set of masses that were used for each metabolite. However, as metabolites with similar selective masses and RIs can be present in metabolite libraries it is necessary to reduce redundancy. *TargetSearch *does this by selecting peaks for which the RI gap is smaller than a defined cut-off and computing the Pearson correlation between them. When two or more metabolites within such a time-group are tightly correlated only the one with more correlated masses is retained. The potential ambiguity is given in the metabolite identity information. This mostly occurs with large, comprehensive libraries which contain many metabolites that elute at the same time and are thus not distinguishable with any approach, or metabolites that are not in the samples, but whose RI and selective masses match one metabolite that indeed is there. Such ambiguity is largely avoided in the case that a curated or more selective library is used.

To aid objective metabolite identification a spectrum similarity score per metabolite is given in the information section. The score includes either all library masses or only the masses that correlate. The score is calculated according to [25]. Let *I*_*m *_and *J*_*m *_the intensities of mass trace *m *of sample spectrum and reference spectrum, respectively. Then the score *S *is:(1)

where *N *is the total number of masses. If one mass is missing, then the intensity of that mass is zero.

### Output

At the end, three matrices are given. One contains the so called metabolite information. This consists of metabolite names; number of correlating masses and the actual correlating mass values, the library RI, found RI and the deviation, spectra similarity score, and the number of samples in which a given metabolite was found. The other two matrices contain the normalised intensities and the RIs, where columns are samples and rows metabolites.

### Graphical user interface

**Figure 2:**
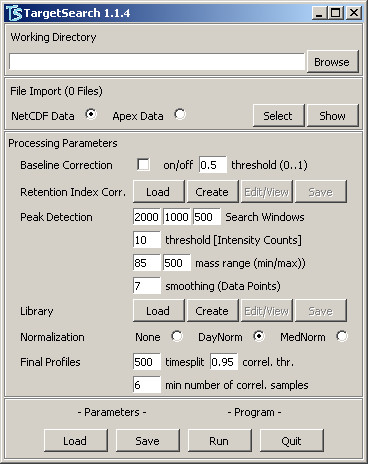
**TargetSearch grahical user interface**. A simple GUI for *TargetSearch*. Here, the user can set all the parameters, import or manually edit the sample files, retention markers definition and library for and run the analysis in one go.

In order to facilitate the use of *TargetSearch *for users unfamiliar with R, we provide a simple to use graphical user interface (GUI). A screenshot can be seen in Figure 2. Many parameters that would be set by calling *TargetSearch *functions can be set here before running the complete analysis. After the analysis is finished, the results are stored in a set of text files that contain the matrices described in the section above. To invoke the GUI, use the R-command *TargetSearchGUI()*.

## Results and Discussion

In order to validate and demonstrate our method, we performed pre-processing analyses of both a standard mixture experiment and a biological example dataset. The standard mixture dataset allows the performance of *TargetSearch *to be objectively assessed by comparing the results against the known concentration ranges, whilst the analysis of a biological dataset and the resulting comparison to manual data curation provides an example of the performance on an actual experiment. The standard mixture experiment is described in the example in order to demonstrate the analysis workflow, whilst only the interpretation and comparison of results is presented for the biological dataset.

### Standard mixture experiment pre-processing

The following section describes the analysis of the standard mixture experiment containing 27 samples using *TargetSearch*. It is worth noting that these samples were measured in another laboratory (RIKEN Plant Science Center, Japan), which shows that *TargetSearch *can work for other data and not only for data generated by our GC-MS platform. Each sample consisted of a mixture of 44 chemical standards at different proportions that were measured as described by Kusano et al. [26]. All chemicals used for GC-TOF/MS analysis were purchased from Wako (Tokyo, Japan) or Sigma-Aldrich (Tokyo, Japan). See Additional file 1 for a detailed list of the standard proportions. The chromatograms are available for download via DROP Met at http://prime.psc.riken.jp. We provide a detailed R-script (Additional file 2) to perform the analysis of the 27 samples. See the *TargetSearch *vignette or the user manual for further options not covered here.

### Data pre-processing example

The first step in GC-MS data pre-processing is to import the chromatogram NetCDF files to *TargetSearch*. In order to do that, the user must provide the sample ("samples.txt") and retention index ("rimLimits.txt") markers definitions, as described in the *user input *subsection (see Additional files 3 and 4). The following R-code shows how both definition files are imported.

> samples <- ImportSamples("samples.txt", RIpath=".", CDFpath=".")

> rimLimits <- ImportFameSettings("rimLimits.txt", row.names = 1)

The parameters set the directories where the chromatograms are located and where the extracted peak list files will be saved. After that, the user needs to perform baseline correction and peak identification. Obviously, baseline correction is needed here because these chromatograms have not already been corrected. Otherwise this step is optional.

The *RIcorrect *function performs both algorithms and is the most time consuming step of the whole pre-processing analysis. In this stage it is possible to specify several parameters: *massRange *parameter limits the mass values (*m/z*) that will be included in the analysis, smoothing *Window *parameter is the number of raw data points that will be averaged in order to remove noise, *IntThreshold *defines the minimum apex intensity to be listed, *baseline *indicates that baseline correction will be performed, and *baseline.opts *is a list object that contains the parameters passed to the baseline algorithm.

> RImatrix <- RIcorrect(samples, rimLimits, massRange = c(75,550), + Window = 15, IntThreshold = 50, baseline = TRUE, baseline.opts = + list(threshold = 0.5))

The function returns a matrix (*RImatrix*) which contains the retention times of the RI markers (rows) found in every sample (columns) and creates a peak list tab delimited file per chromatogram as described in the peak identification section.

This took around 13 minutes to run on an Intel^® ^Core™2 CPU, 2.13 GHz, 2Gb of RAM computer. It should be mentioned that performing baseline correction doubles the running time in comparison to the running time of already corrected chromatograms, so is very useful to select baseline correction when exporting the chromatograms with the vendor software.

**Figure 3:**
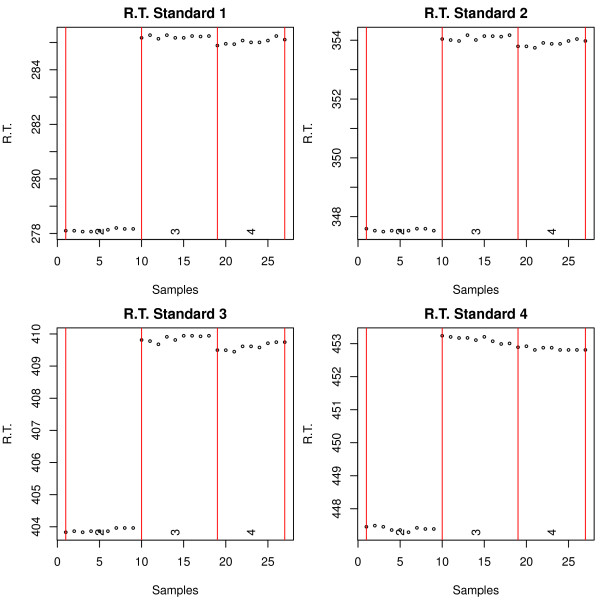
**Retention index markers report**. Retention time of the first four retention index markers in the standard mixture experiment. Samples and retention times are represented in the *x *and *y*-axis, respectively. Different days of measurement are indicated by numbers 2, 3 and 4. A so-called day effect is observed between days 2, and 3 and 4, manifested as a retention time shift.

As mentioned before, an essential step is to look for RI markers outliers. This can be easily done by the function *FameOutliers *which creates a separate plot for each marker and reports whether or not outliers have been found (Figure 3). In this example, we did not find any outliers, but we did observe a strong so-called "day effect" when comparing the measurement day number 2 against days 3 and 4.

outliers <- FAMEoutliers(samples, RImatrix) Outliers Report:

===============

No outliers were found.

The next step is to import the library file (Additional file 5), which is done by the function *ImportLibrary*. Useful options are *TopMasses*, which takes the top *N *most intense masses from the reference spectrum to be searched for, and *RI_dev *sets the RI deviations. The RI deviations can be equal for all metabolites (a vector of length three), or individual ones (a matrix of three columns and as many rows as metabolites) for fine tuning, i.e., it is possible to set specific RI deviations per metabolite.

> lib <- ImportLibrary("metabLibrary.txt", RI_dev = c(15,8,3), TopMasses = 20)

The metabolite search is performed in three steps as explained in the *library search *subsection, where a smaller window is used in every following step. Function *medianRILib *searches the average RI of the selective masses and returns an updated library object. Function *sampleRI *searches for the selective masses in the samples using the updated average RIs and returns a matrix where each element (*i, j*) represents the average RI of metabolite *i *in sample *j*. Finally, function *peakFind *searches again for the top masses and returns the apex intensities and RIs. The three step search is performed by the following R-code.

> lib <- medianRILib(samples, lib)

> corRI <- sampleRI(samples, lib)

> peakData <- peakFind(samples, lib, corRI)

The object *peakData *object has two slots containing all apex raw intensities and RIs of all the masses that were searched for in matrix form. The apex matrix can in principle be used to perform further statistical analysis, by either using R or other statistical tools. In addition, we provide two functions, *Profile *and *ProfileCleanUp*, that create a metabolite profile out of the raw apex data.

> metabProfile <- Profile(samples, lib, peakData)

> finalProfile <- ProfileCleanUp(metabProfile, timeSplit = 2, r_thresh = 0.95)

The first function makes the metabolite profile and the second one looks for possible ambiguities, which are controlled by the parameters *timeSplit *and *r_thresh*. They define the RI gap cut-off that is used to look for metabolites whose RI distance is less than this gap and the correlation threshold, respectively. A conceptually similar approach so-called "time groups" is implemented in the TagFider software [15]. A summary of the obtained metabolite profile is shown in Table 1 (see Additional file 6 for the full profile). We could identify 43 of the 44 standards. All metabolites have at least seven correlating masses, except maltose that has only four. The similarity score (1) was over 790 for every metabolite except histidine (443), fructose-6-phosphate (661) and maltose (716). Only one ambiguous assignment was found: ribose and arabinose. This may have happened because both metabolites have very similar RIs, the same selective masses and similar spectra. One way to resolve such ambiguity can be setting smaller RI deviations and/or using different selective masses.

**Table 1:** Summary of the standard mixture metabolite profiling.

Name	Mass count	RI	Score	RI dev	Sample Count	Corr Coef
Glycolic acid	20	1056.8	977	6.2	27	0.874
Alanine	19	1090.0	950	0.7	27	0.968
Valine	20	1213.5	960	-4.7	27	0.982
Leucine	7	1267.9	995	-1.8	27	0.988
Isoleucine	7	1289.7	993	-1.1	27	0.990
Proline	20	1299.2	869	-1.2	27	0.987
Nicotinic acid	13	1301.5	959	-1.7	27	0.986
Glycine	15	1305.0	790	-0.5	27	0.976
Fumaric acid	20	1346.1	880	0.1	27	0.982
Serine	21	1353.2	929	0.1	27	0.991
Threonine	21	1380.1	917	-0.6	27	0.997
Glutaric acid	20	1402.7	961	-1.7	27	0.947
Alanine, beta-	21	1429.8	929	-5.0	27	0.994
Homoserine	20	1443.7	949	-1.5	27	0.820
Aspartic acid	20	1510.0	915	-0.2	27	0.983
Methionine	20	1519.2	922	-4.7	27	0.985
Butyric acid, 4-amino-	19	1530.0	967	-3.5	27	0.950
Glutaric acid, 2-oxo-	19	1567.0	955	1.2	27	0.951
Phenylalanine	21	1635.3	936	-6.0	27	0.982
Ribose | Arabinose	20 | 20	1662.7 | 1662.7	915 | 942	4.2 | -4.7	27	0.829
Suberic acid	20	1695.0	867	-0.3	27	0.985
Aconitic acid	19	1736.9	977	3.6	27	0.919
Shikimic acid	15	1791.4	975	1.1	27	0.986
Citric acid	17	1806.1	920	-1.6	27	0.948
Isocitric acid	9	1804.6	909	0.0	27	0.828
Arginine	16	1817.8	826	-3.4	27	0.993
Quinic acid	14	1845.0	882	-1.8	27	0.896
Fructose	22	1854.3	922	1.9	27	0.867
Mannose	21	1869.1	908	-0.1	27	0.689
Lysine	20	1912.0	856	0.3	27	0.993
Histidine	17	1914.1	443	-2.5	27	0.983
Galacturonic acid	22	1926.8	948	0.7	27	0.783
Tyrosine	21	1934.0	955	-1.4	27	0.988
Sinapic acid	20	2056.1	962	-2.4	27	0.868
Inositol, myo-	21	2085.0	911	-1.2	27	0.959
Caffeic acid	21	2133.0	957	0.2	27	0.913
Phytol	21	2169.7	954	-0.6	27	0.745
Tryptamine	19	2244.2	864	-14.8	27	0.838
Fructose-6-phosphate	12	2286.3	661	6.5	27	0.738
Cystine	13	2290.0	860	0.6	27	0.971
Glucose-6-phosphate	21	2301.9	958	5.2	27	0.866
Maltose	4	2723.7	716	3.4	27	0.741
Trehalose	16	2729.8	853	0.3	27	0.949

The summarised metabolite profile of the chemical standards (see Additional file 6 for full information). Name: metabolite name; Mass count: the number of correlating masses; RI: averaged RI; Score: spectrum similarity according to (1); RI dev; RI deviation from the expected RI; Sample Count: the number of samples in which the metabolite was found; Corr Coef: the correlation coefficient between metabolite input concentration and abundance estimation.

Since the chemical standards were measured using different proportions (Additional file 1), we examined the correlation between the abundance estimation with the input concentration in order to further confirm the *TargetSearch *results. There is a clear correlation for all the metabolites (Table 1, Additional file 7). Most of the metabolites (29) had a correlation coefficient greater than 0.9 and the lowest coefficient was 0.69. Here we showed that with little effort we can perform a metabolite analysis that can be used with little manual curation needed (only 4 of 44 has to be manually checked).

## Visualisation

**Figure 4:**
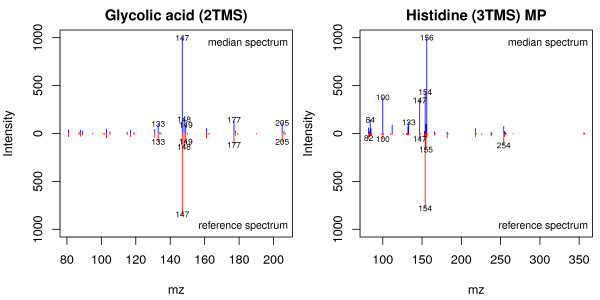
**Comparison between identified metabolite spectra and reference spectra**. Comparison example between the averaged (blue) and reference spectra (red) of glycolic acid (right) and histidine (left). This type of comparisons can be used to assess the quality of individual identified peaks. In this example, histidine may need a closer inspection to elucidate what causes the difference.

After GC-MS pre-processing, the user can visually compare the averaged metabolite spectra with the reference spectra. Our software include two functions to do so. *plotSpectra *and *plotAllSpectra*. They plot a spectra contrast between averaged metabolite spectra, obtained by computing the median intensity of every mass across the samples, and reference spectra for a given metabolite (Figure 4). This allows the user to quickly validate the results by visually inspecting the contrasted spectra. In addition, the metabolite spectra can also be exported as a MSP format file (function *writeMSP*), which can be imported into NIST for further comparisons against other metabolite libraries.

We additionally provide a function to visualise chromatographic peaks in a given sample, which may also help to identify metabolites. See the *TargetSearch *vignette for examples.

### Biological dataset pre-processing

The biological dataset consisted of wild-type Col-0 *Arabidopsis thaliana *seedling samples. Three week old seedlings, grown on solid MS medium with 1% sucrose, were kept for 4h under either continuous light or darkness. Metabolites were extracted from pools of seedlings in a total of four biological replicates. Extraction and derivatisation of metabolites from leaves using GC-MS were performed as outlined by Lisec et al. [24]. GC-MS data were obtained using an Agilent 7683 series autosampler (Agilent Technologies GmbH, Waldbronn, Germany), coupled to an Agilent 6890 gas chromatograph - Leco Pegasus 2 time-of-flight mass spectrometer (LECO, St. Joseph, MI, USA). Identical chromatogram acquisition parameters were used as those previously described by Weckwerth et al. [27].

A similar pre-processing analysis as the one described for the standard mixture dataset was performed. We used a fatty acid methyl esters (FAMEs) as RI marker standards (Additional file 8) and an in-house reference library composed of 153 metabolites (Additional file 9). This library was manually curated and in addition to known metabolites includes several unknown metabolites that have been observed in previous experiments.

After running *TargetSearch*, we manually compared the final profile with the deconvoluted peak profiles obtained with LECO (see Additional file 10 for full profile with manual annotations). The profile list contained 138 entries in total, of which 131 metabolites were unambiguously assigned. The 7 ambiguous assignments corresponded to 14 metabolites (2 metabolites per entry). The remaining 8 metabolites (of the 153) were neither present in the final profile nor in the chromatograms as confirmed by manual inspection. Based on our previous experience, we routinely only consider metabolites to be present if at least 3 correlating masses at the correct RI are identified (this excludes the duplicate isotope pairs that are often observed to correlate). Taking this into account, we would consider 101 metabolites to be identified in this experiment. We thus checked these manually and found that 96 were correctly assigned (these were all assigned a similarity score above 600), 1 ambiguity was wrongly resolved, i.e., the correct metabolite was not the one suggested; 1 ambiguity could not be resolved manually, due to similar RIs and reference spectra; 1 metabolite was not found in the chromatograms; and 2 metabolites were not found but 2 peaks (unknowns) were found at their expected retention time. The later could be anticipated in the profile since the similarity score reported by *TargetSearch *was below 400.

**Figure 5:**
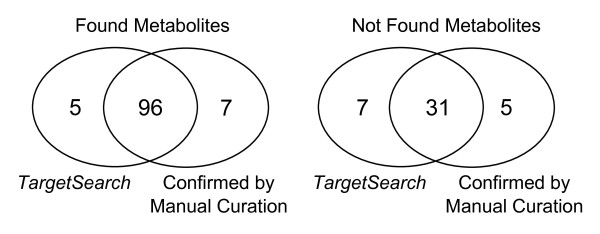
**Comparison between identified metabolites by TargetSearch and manual curation**. The venn diagrams show the number of metabolites considered to be either present or not present by *TargetSearch *in comparison to the manual curation. Left hand side: 96 metabolites identified by *TargetSearch *that were confirmed by manual curation. Right hand side: 31 metabolites not identified by *TargetSearch *and confirmed by manual curation. Both diagrams: 5 metabolites found by our software but not confirmed by manual curation, whilst 7 metabolites were not reported by *TargetSearch *but were present in the chromatograms.

Although we would not routinely work with the remaining 37 entries (38 metabolites in total) that did not meet our correlating masses minimum, we additionally manually inspected these. Their classification was as follows: 31 metabolites, including 1 ambiguity, were not found in the chromatograms; 5 metabolites were only found in a few of the chromatograms at low abundance and 2 metabolites were present but overloaded. In summary, 96 of 101 metabolites found by *TargetSearch *were correctly assigned. Similarly, 31 of 38 metabolites that we considered to have too few correlating masses to be present were manually confirmed not to be present in the chromatograms. Just 5 metabolites were found by our software but could not be confirmed to sufficient confidence by manual curation, whilst 7 metabolites were not reported by *TargetSearch *but were present in the chromatograms. This gives over 91% of assignment accuracy, counting the true positives and true negatives ratio. The results are illustrated in Figure 5.

Based on this example analysis and our experience with several other biological datasets, errors can be made by *TargetSearch *in the following cases: metabolites with similar selective masses and spectra that elute at similar retention times; low abundant metabolites may cause spurious correlations, whilst overloaded peaks often elude the peak detection algorithm or disrupt correlation; finally, metabolites not present in the library that elute in the place where another metabolite is expected, as occurred in two cases in this analysis.

Many of these issues can be solved by changing the parameter definitions and in general the resulting dataset is more objectively linked to these parameters than the decisions involved in a typical manual curation. Metabolite identification issues for example can be solved by increasing the intensity threshold during the peak detection stage, or by changing the correlation cut-off. We also note that most of these errors are inherent to metabolite profiling *per se *rather than being specific to our software and are thus also addressed by other technical aspects of the experiment (e.g. saturation/sample overloading or low-abundance).

### Performance

We developed this package with the intention to process as many chromatograms as possible in a single run and to be fast. To asses performance, we compared the processing speed of *TargetSearch *with other GC-MS pre-processing software: XCMS and Tagfinder. First, we wanted to see what was the maximum number of chromatograms that can be processed by said tools. *TargetSearch *could process our largest experiment - 1025 baseline corrected chromatograms (Caldana et al., unpublished), taking around 6 hours to get a final data matrix (Intel^® ^Core™2 CPU, 2.13 GHz, 2Gb of RAM). However, neither XCMS nor Tagfinder were able to process anywhere near that number of chromatograms with the same computer. We then reduced the number of chromatograms to 200 so we could compare the processing times. *TargetSearch *proved to be much faster than XCMS and Tagfinder. Results are summarised in Table 2.

**Table 2:** Performance comparison of pre-processing analysis using Tagfinder, XCMS and TargetSearch.

	Tagfinder	XCMS	TargetSearch
**Maximum number of samples**	**70**	**645**	***> *1025**

*Pre-processing step*:			
Peak extraction	3442	3634	2779
Peak import	271	-	-
RI correction	255	29	-
Profile building	3050	1740	195
**Total time**	**7018**	**5403**	**2974**
**Total time per sample**	**35.1**	**27.0**	**14.9**

All parameters were set to default in order to obtain the maximum number of samples, but only 1025 samples were available. Times were compared using 200 samples. Before running this analysis, Tagfinder intensity threshold was set to 200 to allow the 200 samples to be imported. Time is expressed in seconds. Peak import is not necessary in XCMS and *TargetSearch*. RI correction is performed together with Peak extraction in *TargetSearch*.

## Conclusions

Here we presented the *TargetSearch *software for the data pre-processing of GC-MS based metabolite profiling experiments. Our method includes a novel strategy to iteratively correct and update retention times and uses efficient criteria for searching and identifying metabolites. This massively reduces the time from data acquisition to biological interpretation, particularly as good quality data can be directly obtained with limited need for manual curation. Another advantage over other software, is the ability to analyse virtually any number of samples, at least over 1000, which otherwise is a major limitation of existing software, whilst maintaining a fast processing speed. We also provide a GUI intended to assist users not familiar with R. Using a dilution mixture dataset we found that *TargetSearch *accurately identified the chemical components and reported abundance estimates which very well reproduced the input concentrations. Using a real biological experiment, the accuracy in terms of automated profiling was over 90%. These results show that *TargetSearch *greatly facilitates the analysis of metabolic profiling data and creates substantial time savings allowing scientists to focus more on the biology under investigation.

## Availability and requirements

Project name: TargetSearch

Project homepage: http://bioconductor.org/packages/2.5/bioc/html/TargetSearch.html

Operating System: Platform independent

Programming language: R, C

Other requirements: R *> *= 2.7

License: GPL version 2 or newer

## Abbreviations

GC-MS: Gas Chromatography-Mass Spectrometry; MST: Mass Spectra Tag; RT: Retention Time; RI: Retention time Index; GUI: Graphical User Interface.

## Authors' contributions

MAH initiated and supervised the project and developed an initial method with HR. ACI improved and developed the majority of the method and the R package. CC tested and contributed to the method. JL contributed R scripts and developed the graphic user interface. MK performed GC-MS analysis. LW discussed and supervised the project. ACI drafted the manuscript, with additions from all authors. All authors read and approved the final manuscript.

## Supplementary Material

Additional file 1 **standard compounds**. The list of standard compounds. Additional columns indicate the concentration in *μ*g/*μ*L that was used in every mixture. Click here for file

Additional file 2 **TargetSearch example R-script**. R-script used in this manuscript to process the standard mixture samples. Click here for file

Additional file 3 **Sample file**. The sample description file needed as input for *TargetSearch*. It contains the list of NetCDF files, the measurement day and the mixture used. Click here for file

Additional file 4 **Retention index marker limits**. The Retention index marker file needed as input for *TargetSearch*. Columns represent the standard name, lower and upper limits, RI, and mass. Click here for file

Additional file 5 **Reference metabolite library**. The reference library which contains the information on the standards that were searched for in the chromatograms. Click here for file

Additional file 6 **Metabolite profile**. The metabolite profile information as given by *TargetSearch*. It contains the metabolite names, number of correlating masses, the mass values, the library RI, the found RI, RI deviation and spectra score. Click here for file

Additional file 7 **Metabolite concentration correlation**. Correlation between the input standard concentration and the obtained metabolite abundance. Every boxplot corresponds to one metabolite as indicated in the plot title. Boxes represent mixtures, mixture concentrations are shown above the box, the *y*-axis is the relative abundance, the expected abundances are indicated by horizontal dashed lines, and the correlation coefficient is displayed at the bottom of the graph. Click here for file

Additional file 8 **Retention index marker limits (biological dataset)**. The Retention index marker file needed as input for *TargetSearch *(Biological Dataset). Columns represent the standard name, lower and upper limits and RI. Click here for file

Additional file 9 **Reference metabolite library (biological dataset)**. The reference library which contains the information on the standards that were searched for in the biological dataset chromatograms. Click here for file

Additional file 10 **Biological dataset profile**. The metabolite profile information as given by *TargetSearch*. It contains the metabolite names, number of correlating masses, the mass values, the library RI, the found RI, RI deviation and spectra score. The first column contains the annotations after manual curation. Click here for file
