# Supplementary material for: TargetSearch - a Bioconductor package for the efficient preprocessing of GC-MS metabolite profiling data
Source: BMC Bioinformatics. 2009 Dec 16;10:428. doi: 10.1186/1471-2105-10-428 (PMC3087348; doi:10.1186/1471-2105-10-428)
Supplement: Additional file 7 — Metabolite concentration correlation. Correlation between the input standard concentration and the obtained metabolite abundance. Every boxplot corresponds to one metabolite as indicated in the plot title. Boxes represent mixtures, mixture concentrations are shown above the box, the y-axis is the relative abundance, the expected abundances are indicated by horizontal dashed lines, and the correlation coefficient is displayed at the bottom of the graph. [file 1471-2105-10-428-S7.PDF]

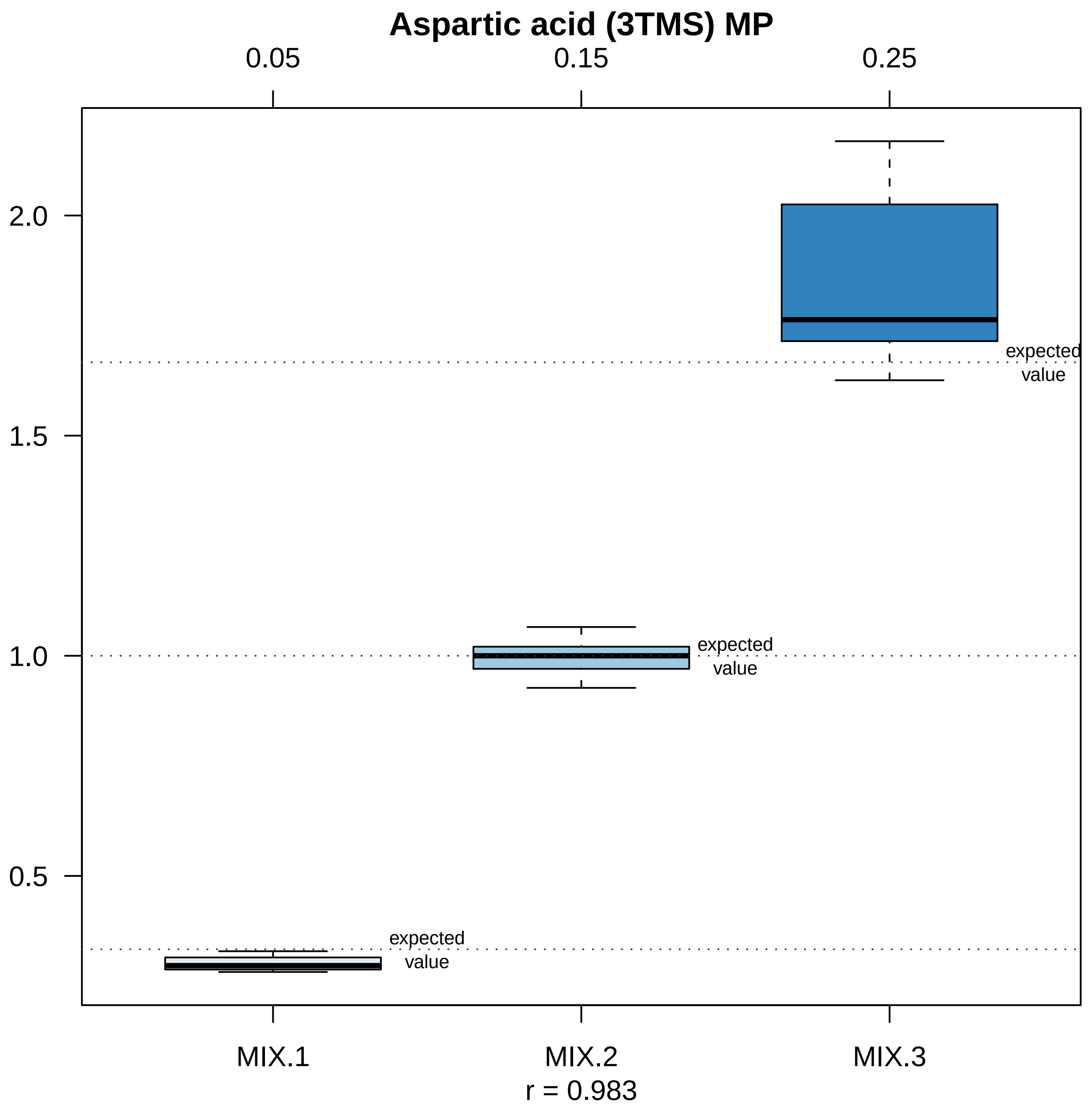
Aspartic acid (3TMS) MP
0.05
0.15
0.25
2.0
1.5
1.0
0.5
expected value
expected value
expected value
MIX.1
MIX.2
MIX.3
r = 0.983

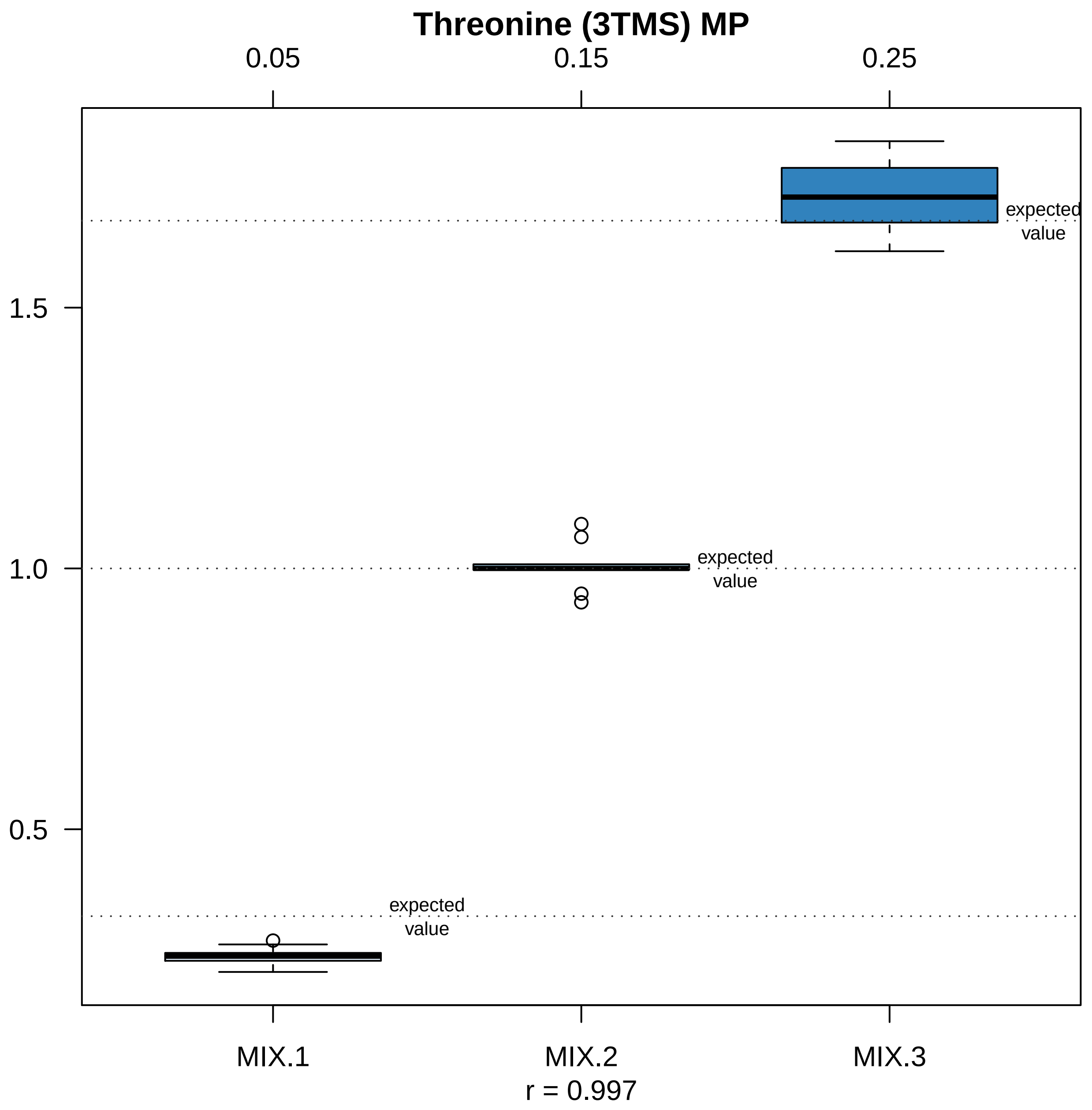

Threonine (3TMS) MP
0.05
0.15
0.25
1.5
1.0
0.5
expected value
expected value
expected value
MIX.1
MIX.2
MIX.3
r = 0.997

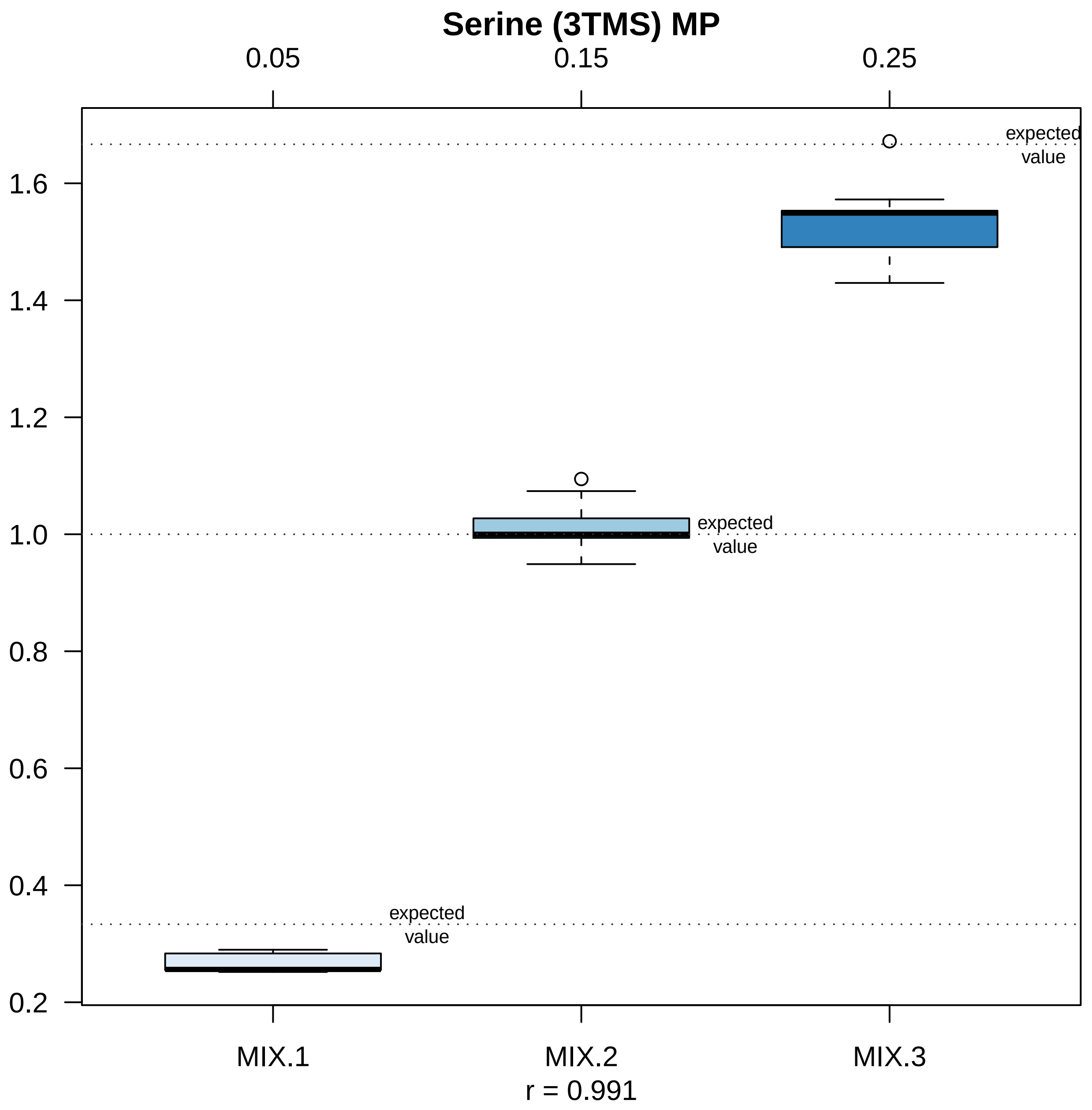
Serine (3TMS) MP
0.05
0.15
0.25
1.6
1.4
1.2
1.0
0.8
0.6
0.4
0.2
expected value
expected value
expected value
MIX.1
MIX.2
MIX.3
r = 0.991

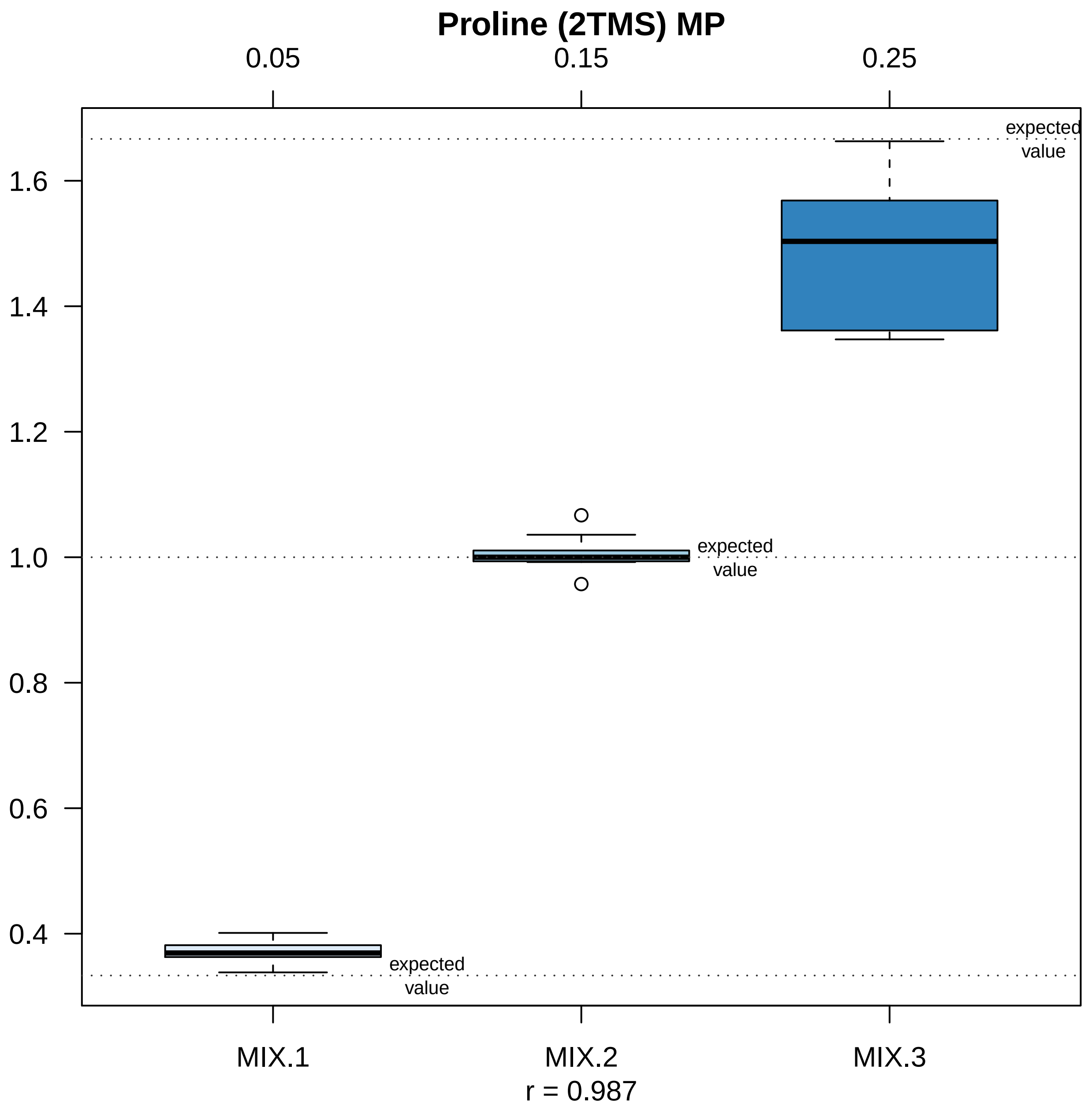
Proline (2TMS) MP
0.05
0.15
0.25
1.6
1.4
1.2
1.0
0.8
0.6
0.4
expected value
expected value
expected value
MIX.1
MIX.2
MIX.3
r = 0.987

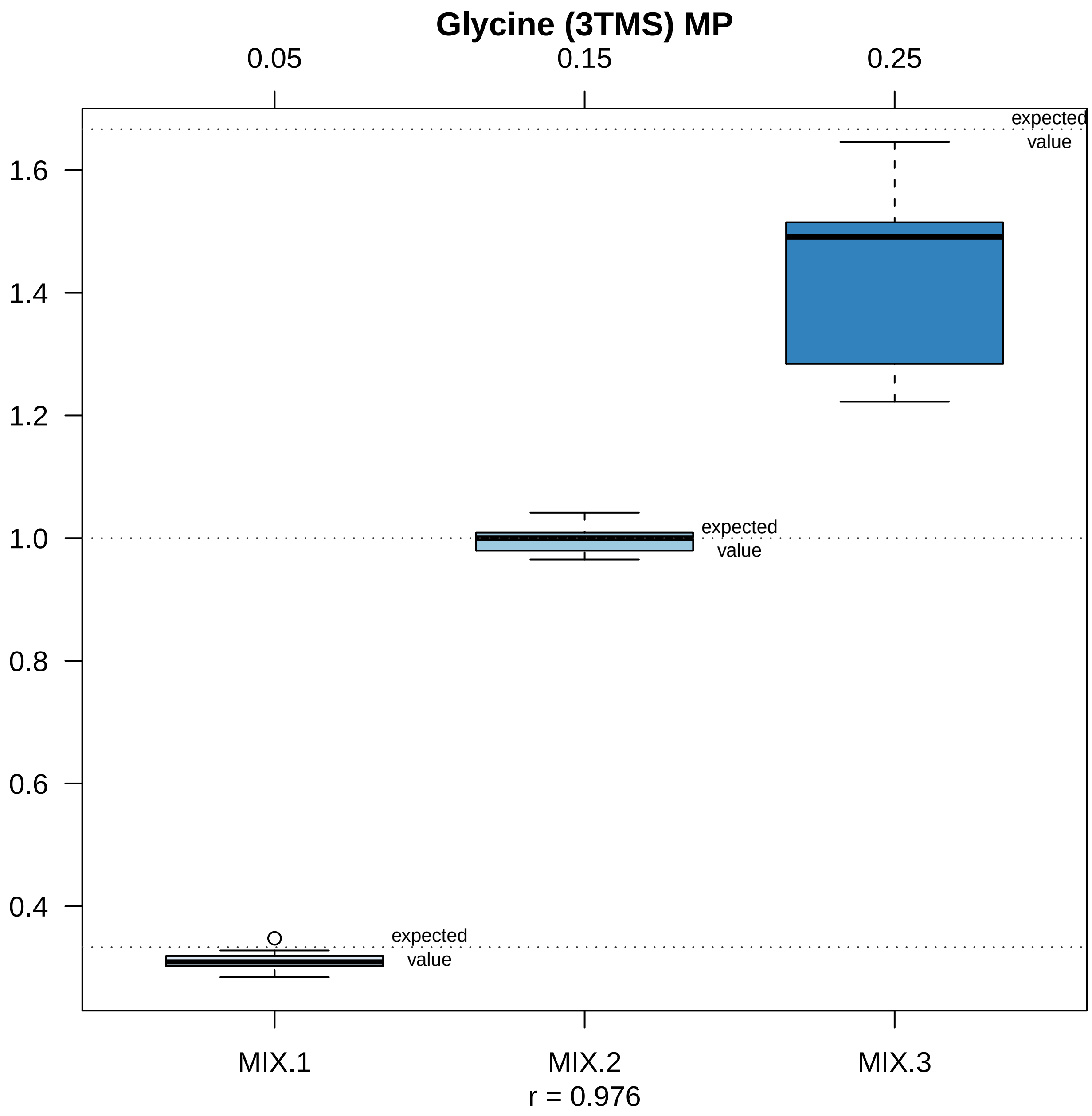
Glycine (3TMS) MP
0.05
0.15
0.25
1.6
1.4
1.2
1.0
0.8
0.6
0.4
expected value
expected value
expected value
MIX.1
MIX.2
MIX.3
r = 0.976

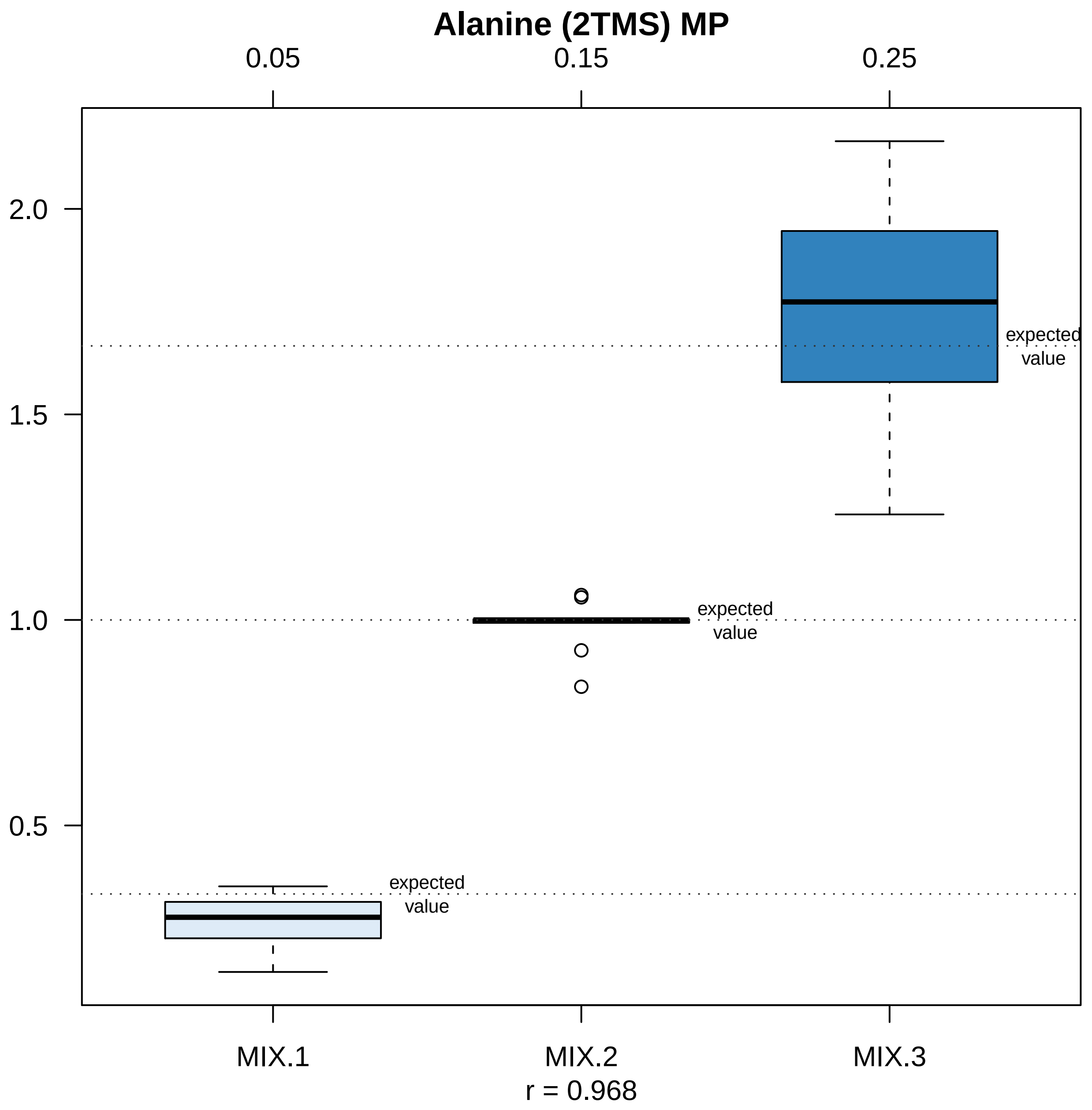
Alanine (2TMS) MP
0.05
0.15
0.25
2.0
1.5
1.0
0.5
expected value
expected value
expected value
MIX.1
MIX.2
MIX.3
r = 0.968

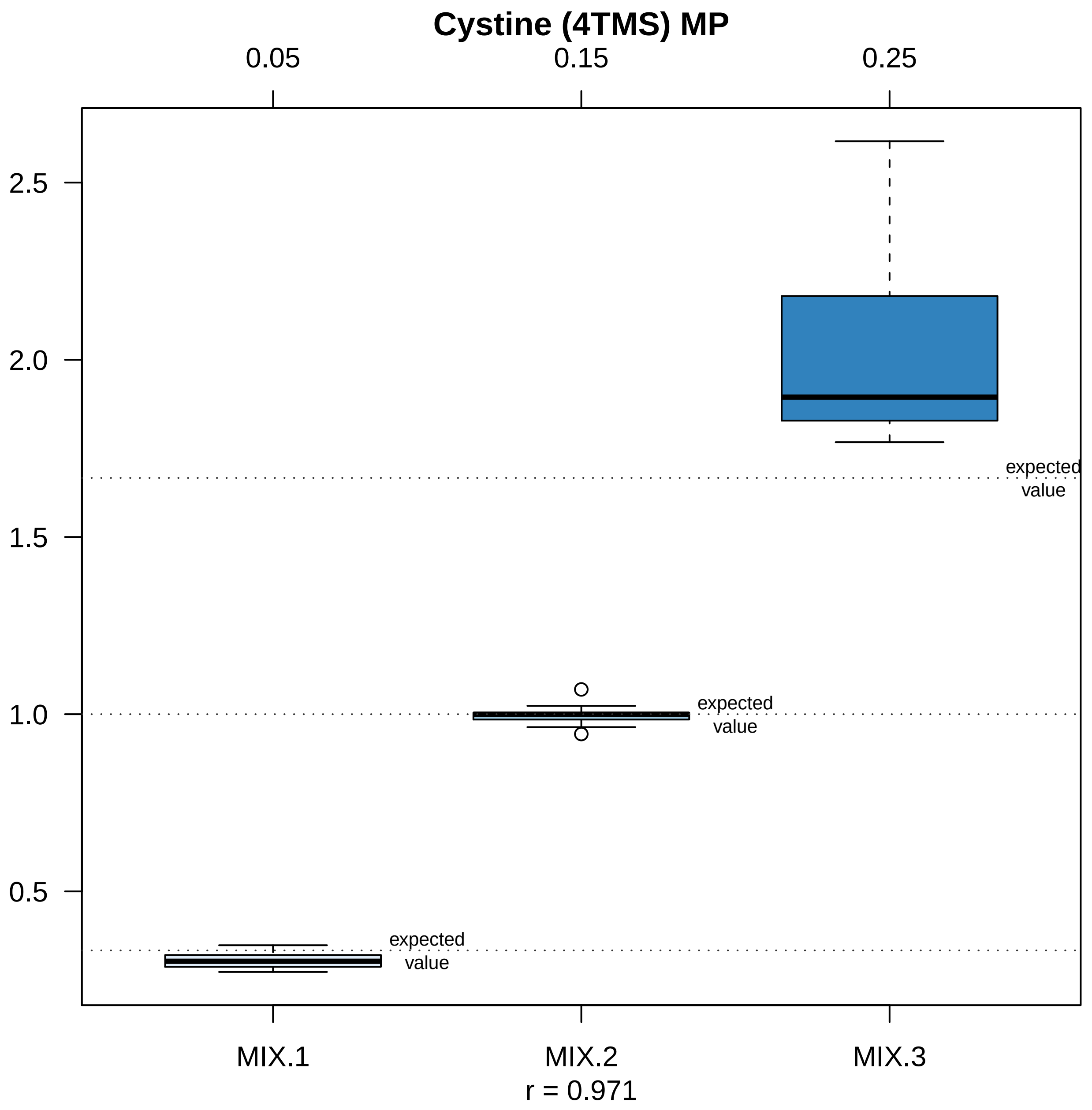
Cystine (4TMS) MP
0.05
0.15
0.25
2.5
2.0
1.5
1.0
0.5
expected value
expected value
expected value
MIX.1
MIX.2
MIX.3
r = 0.971

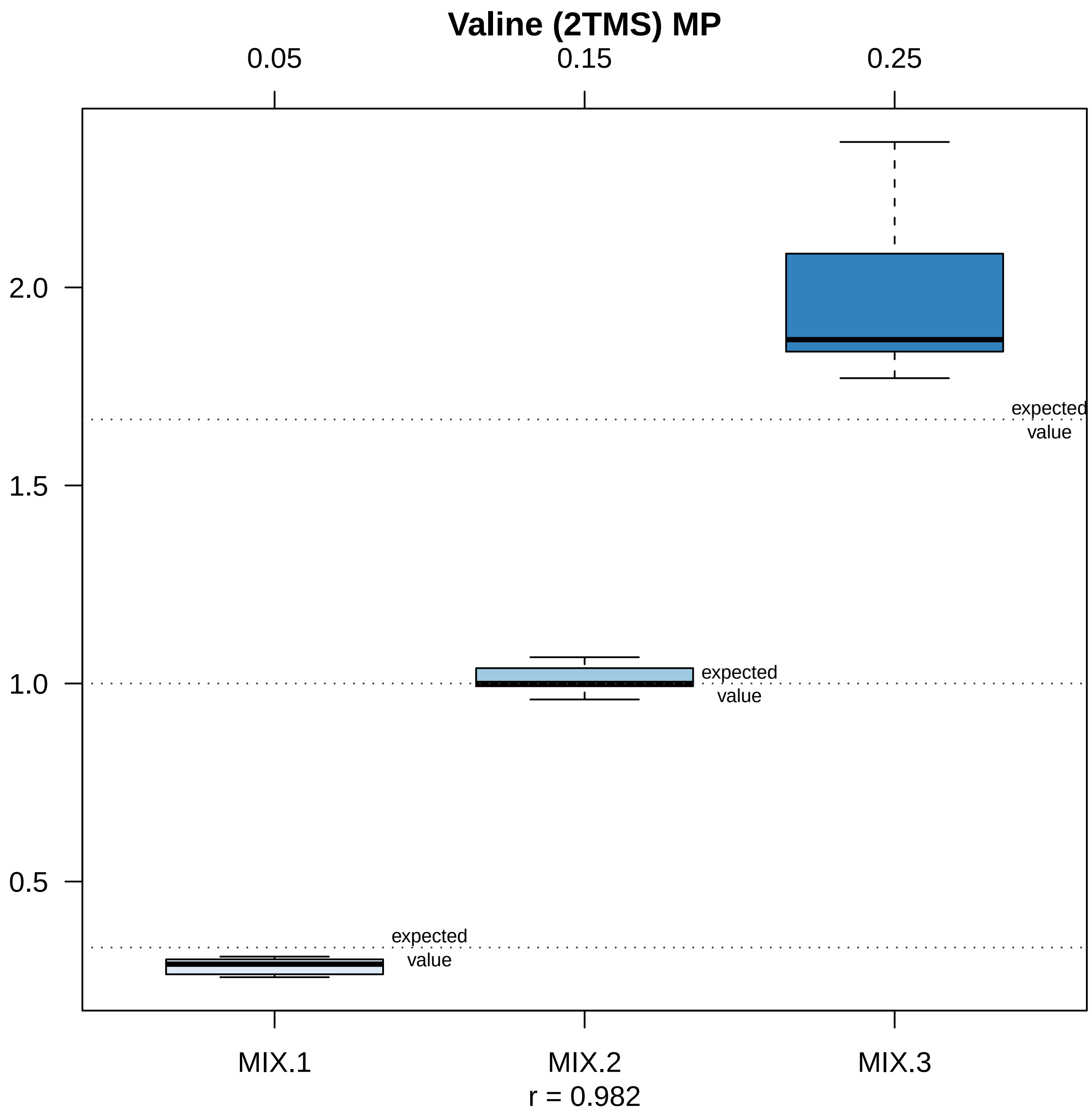
Valine (2TMS) MP
0.05
0.15
0.25
2.0
1.5
1.0
0.5
expected value
expected value
expected value
MIX.1
MIX.2
MIX.3
r = 0.982

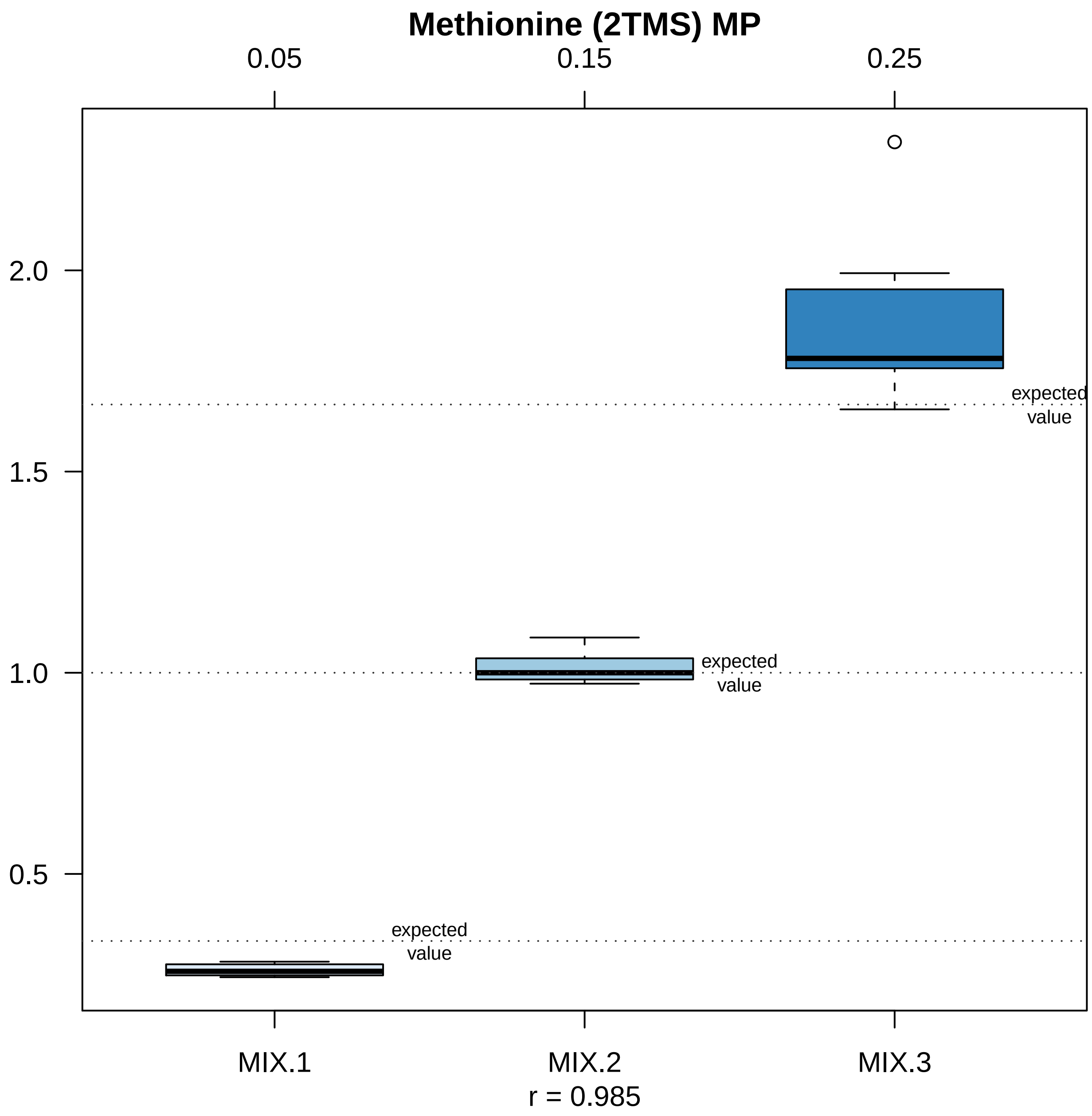
Methionine (2TMS) MP
0.05
0.15
0.25
2.0
1.5
1.0
0.5
expected value
expected value
expected value
MIX.1
MIX.2
MIX.3
r = 0.985

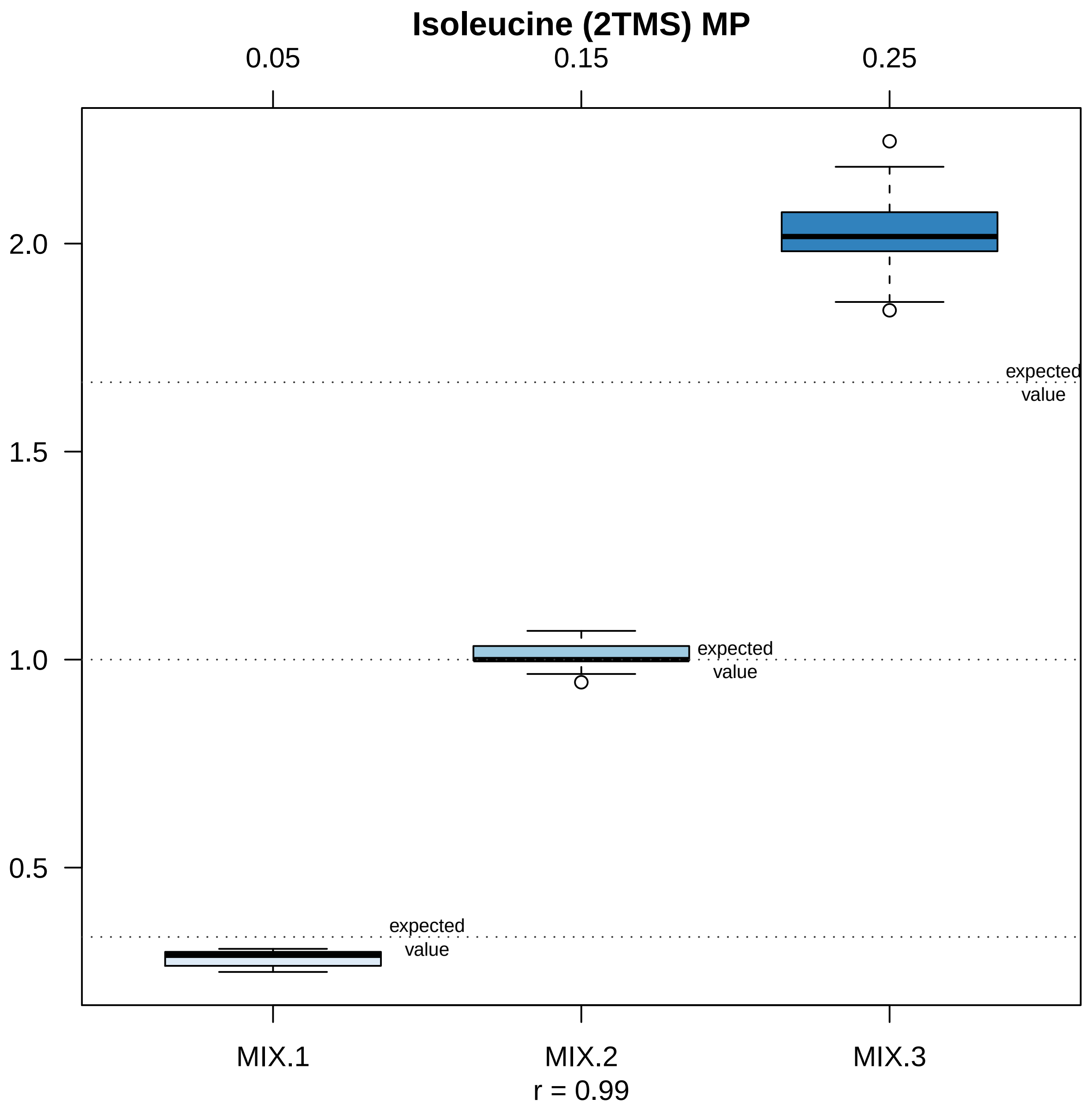
Isoleucine (2TMS) MP
0.05
0.15
0.25
2.0
1.5
1.0
0.5
expected value
expected value
expected value
MIX.1
MIX.2
MIX.3
r = 0.99

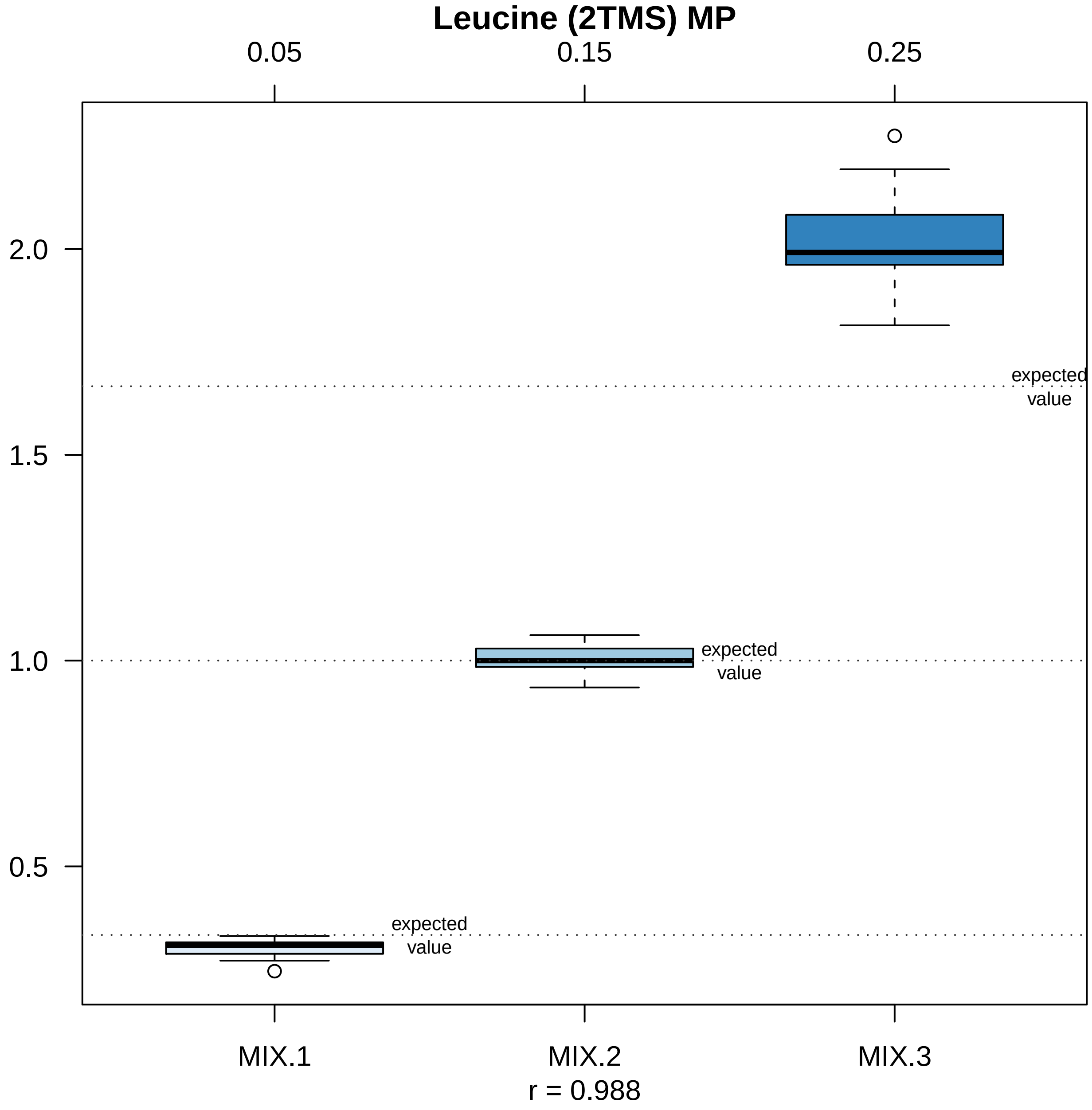
Leucine (2TMS) MP
0.05
0.15
0.25
2.0
1.5
1.0
0.5
expected value
expected value
expected value
MIX.1
MIX.2
MIX.3
r = 0.988

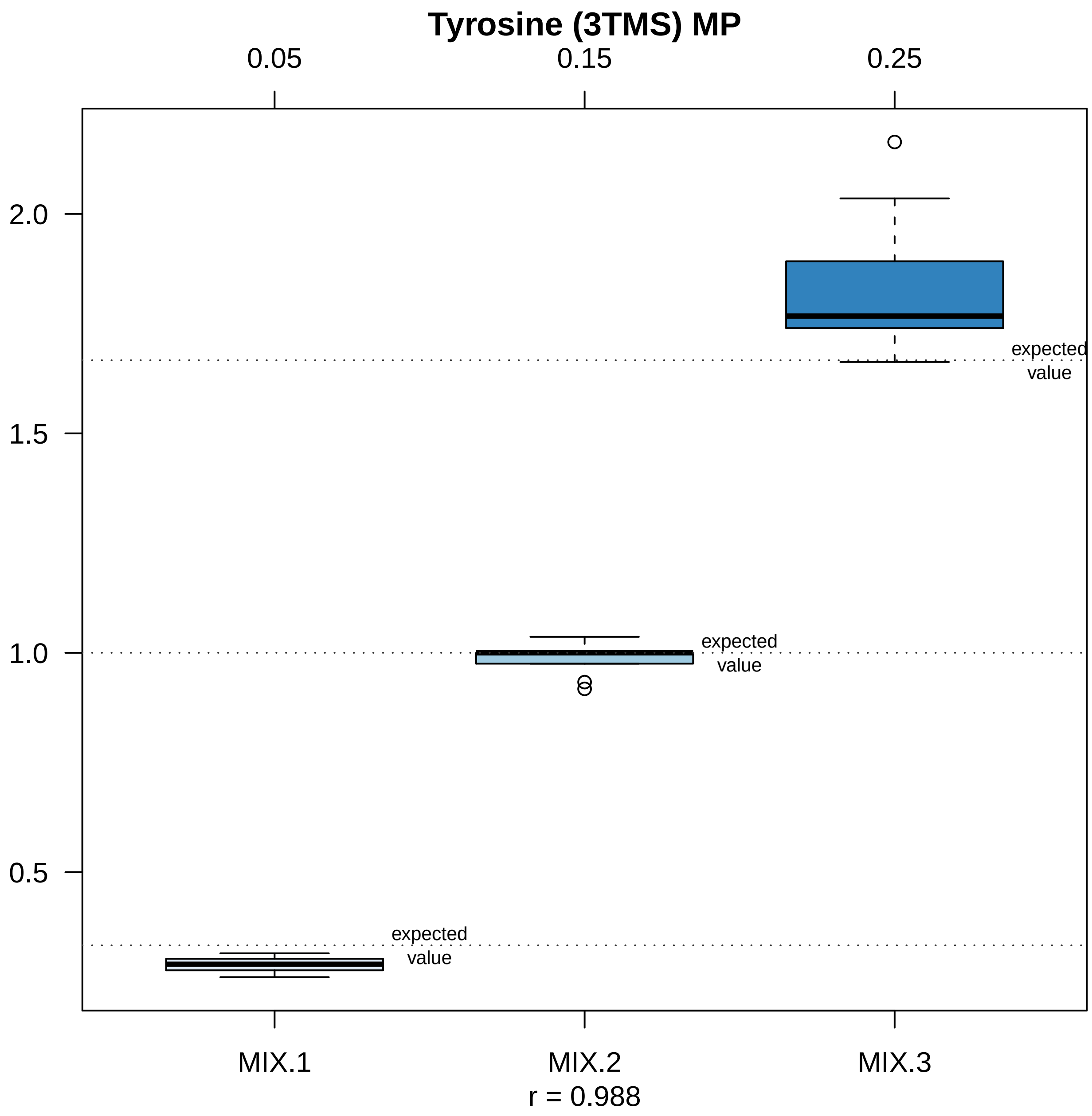

Tyrosine (3TMS) MP
0.05
0.15
0.25
2.0
1.5
1.0
0.5
expected value
expected value
expected value
MIX.1
MIX.2
MIX.3
r = 0.988

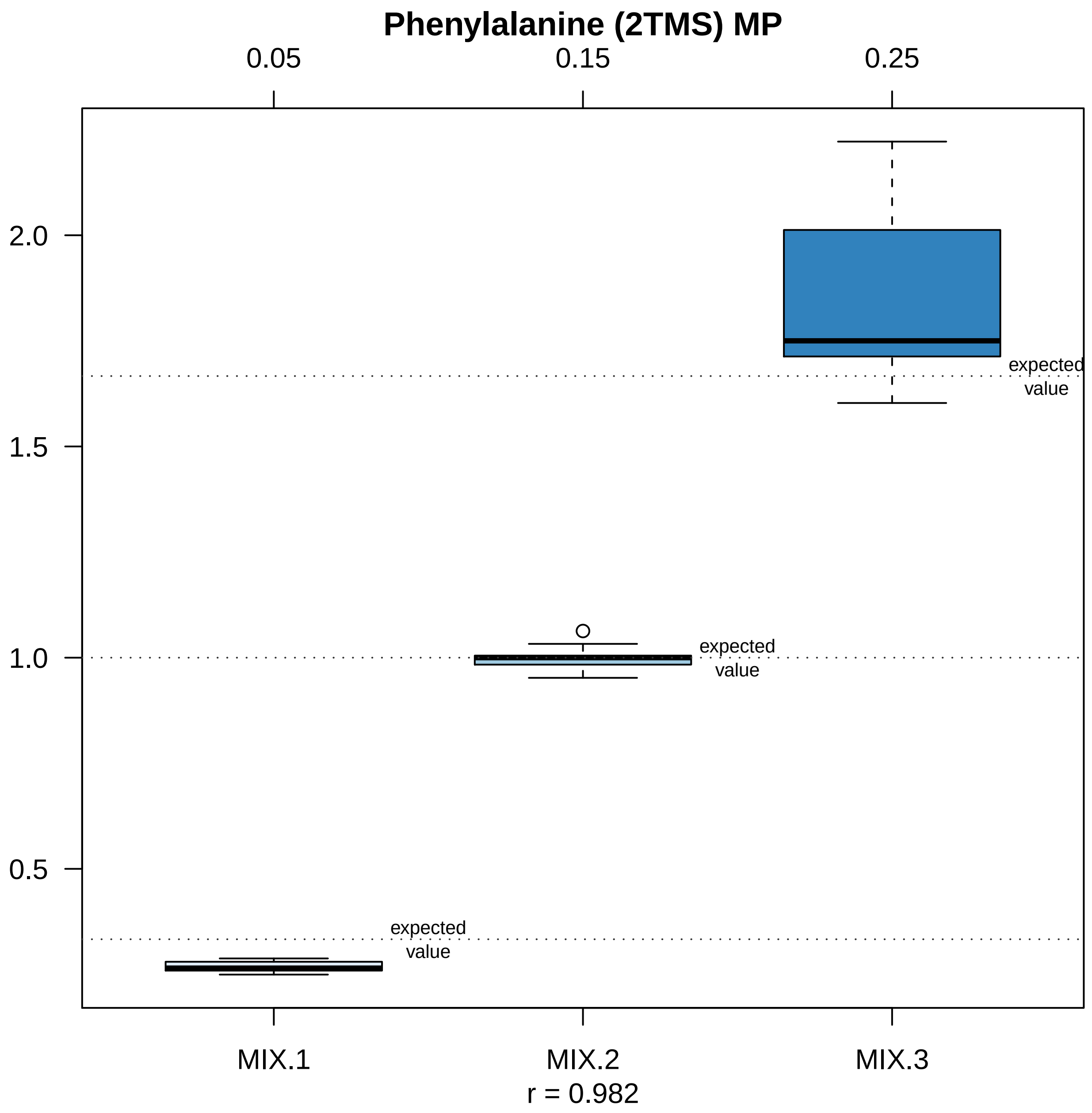
Phenylalanine (2TMS) MP
0.05
0.15
0.25
2.0
1.5
1.0
0.5
expected value
expected value
expected value
MIX.1
MIX.2
MIX.3
r = 0.982

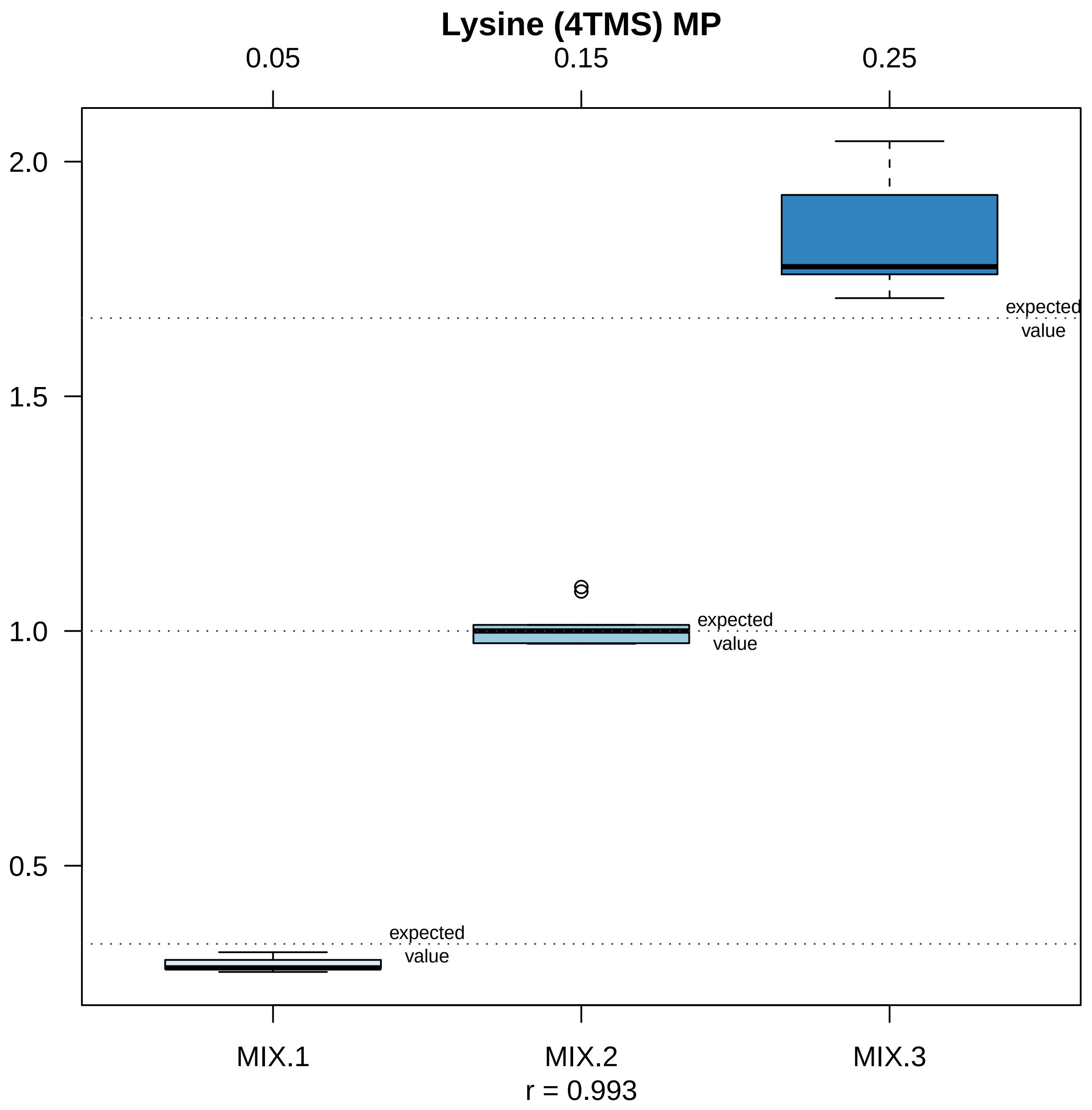
Lysine (4TMS) MP
0.05
0.15
0.25
2.0
1.5
1.0
0.5
expected value
expected value
expected value
MIX.1
MIX.2
MIX.3
r = 0.993

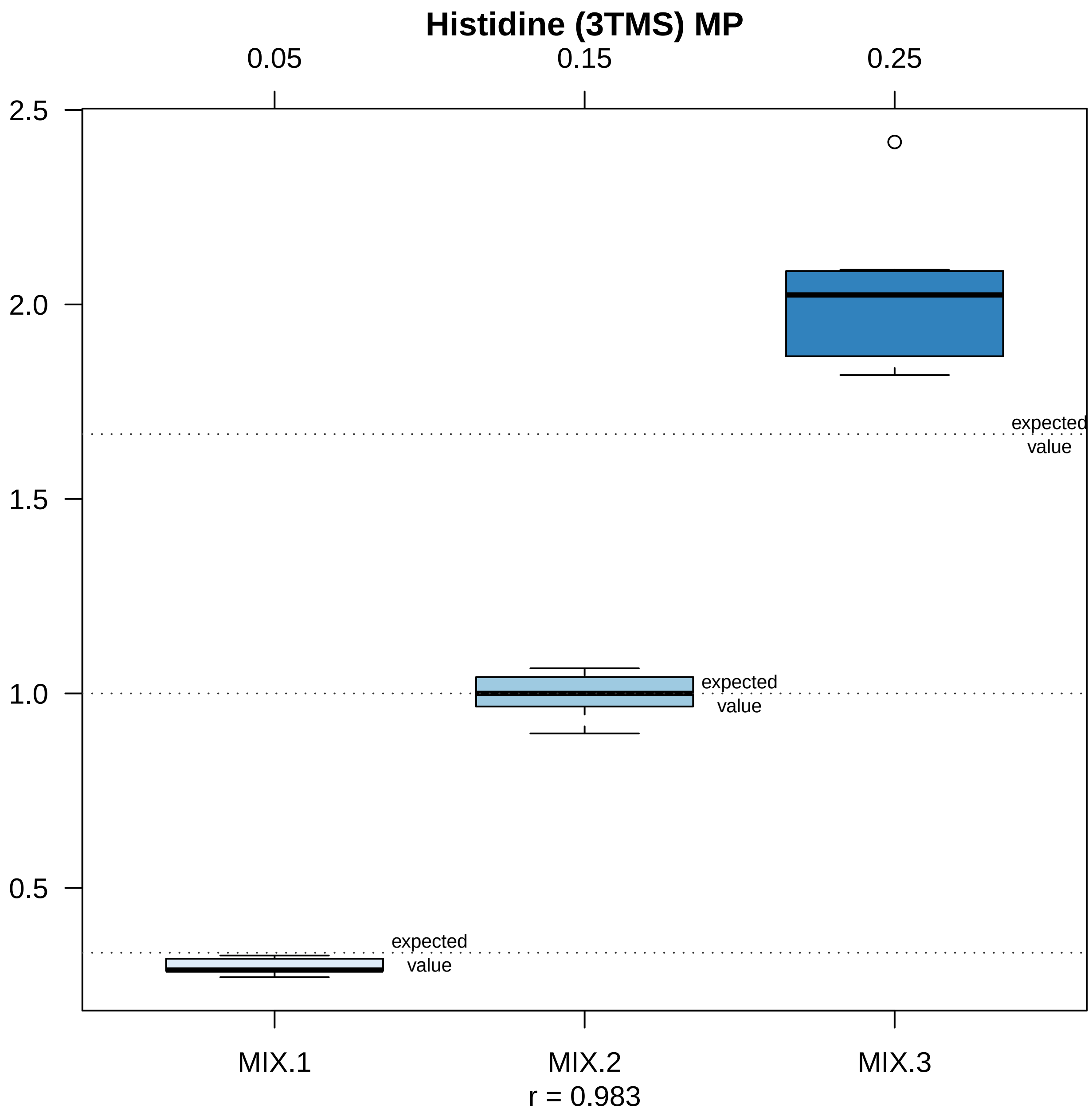
Histidine (3TMS) MP
0.05
0.15
0.25
2.5
2.0
1.5
1.0
0.5
expected value
expected value
expected value
MIX.1
MIX.2
MIX.3
r = 0.983

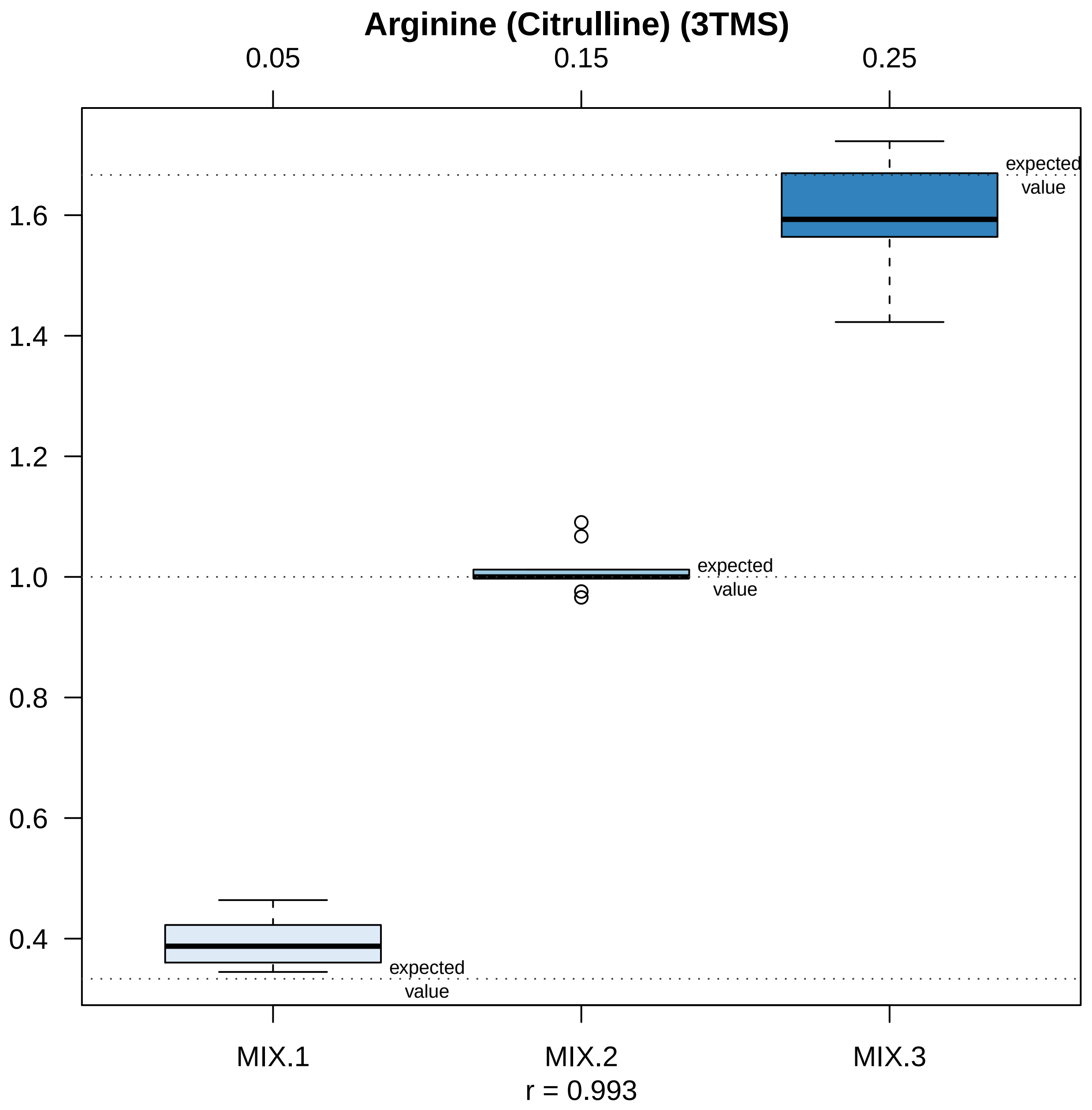

Arginine (Citrulline) (3TMS)
0.05
0.15
0.25
1.6
1.4
1.2
1.0
0.8
0.6
0.4
expected value
expected value
expected value
MIX.1
MIX.2
MIX.3
r = 0.993

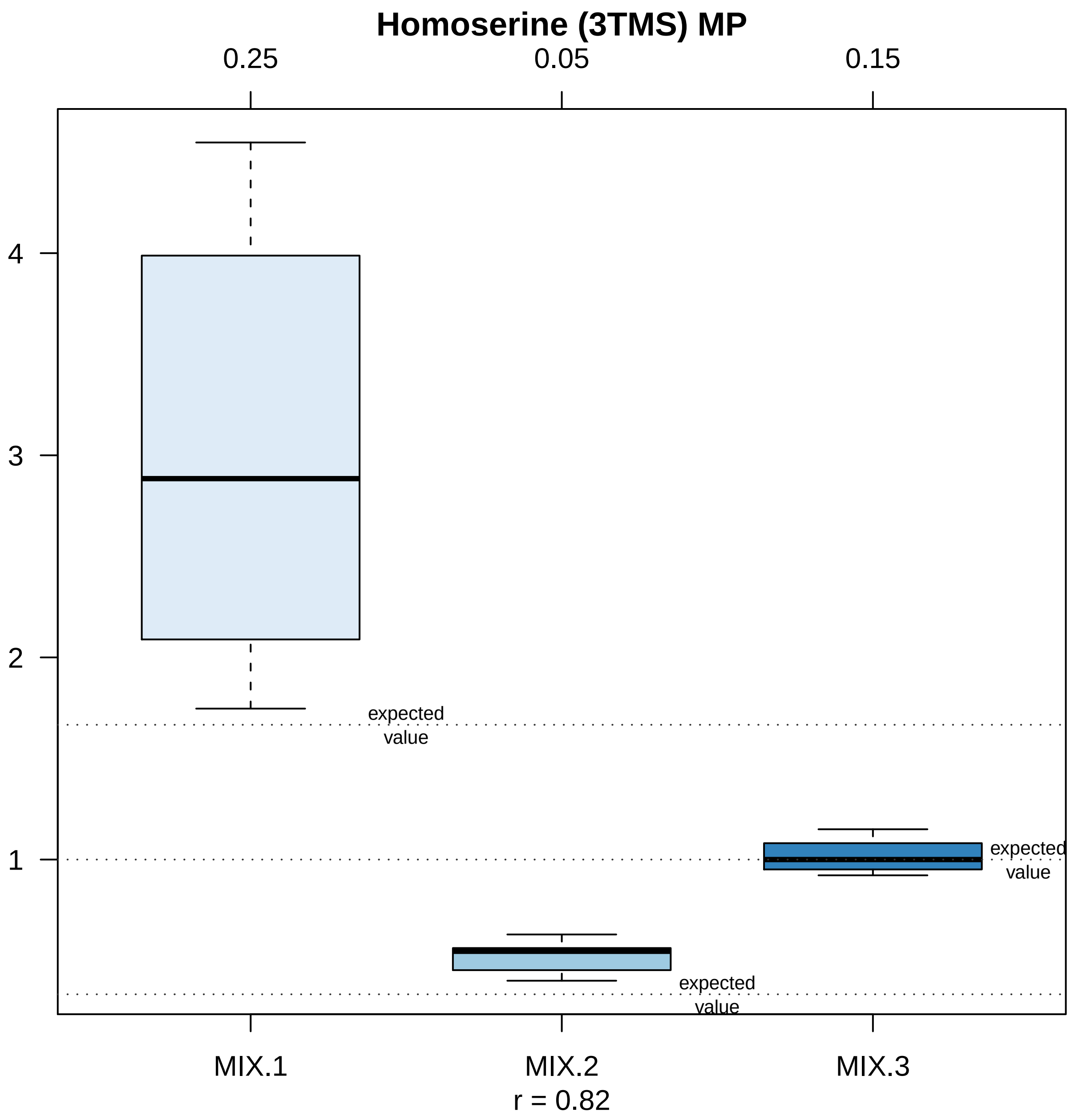
Homoserine (3TMS) MP
0.25
0.05
0.15
4
3
2
1
expected value
expected value
expected value
MIX.1
MIX.2
MIX.3
r = 0.82

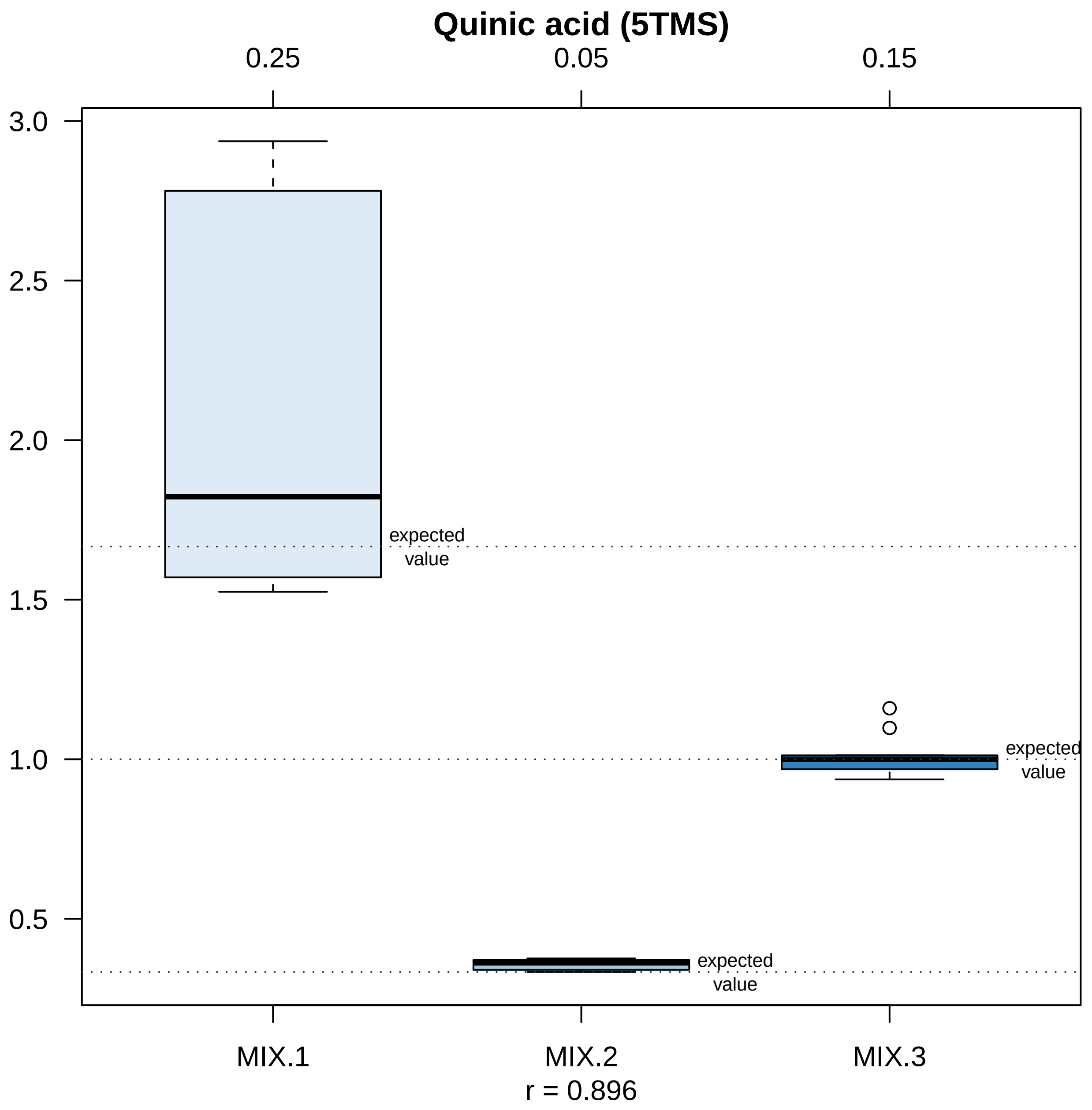
Quinic acid (5TMS)
0.25
0.05
0.15
3.0
2.5
2.0
1.5
1.0
0.5
expected value
expected value
expected value
MIX.1
MIX.2
MIX.3
r = 0.896

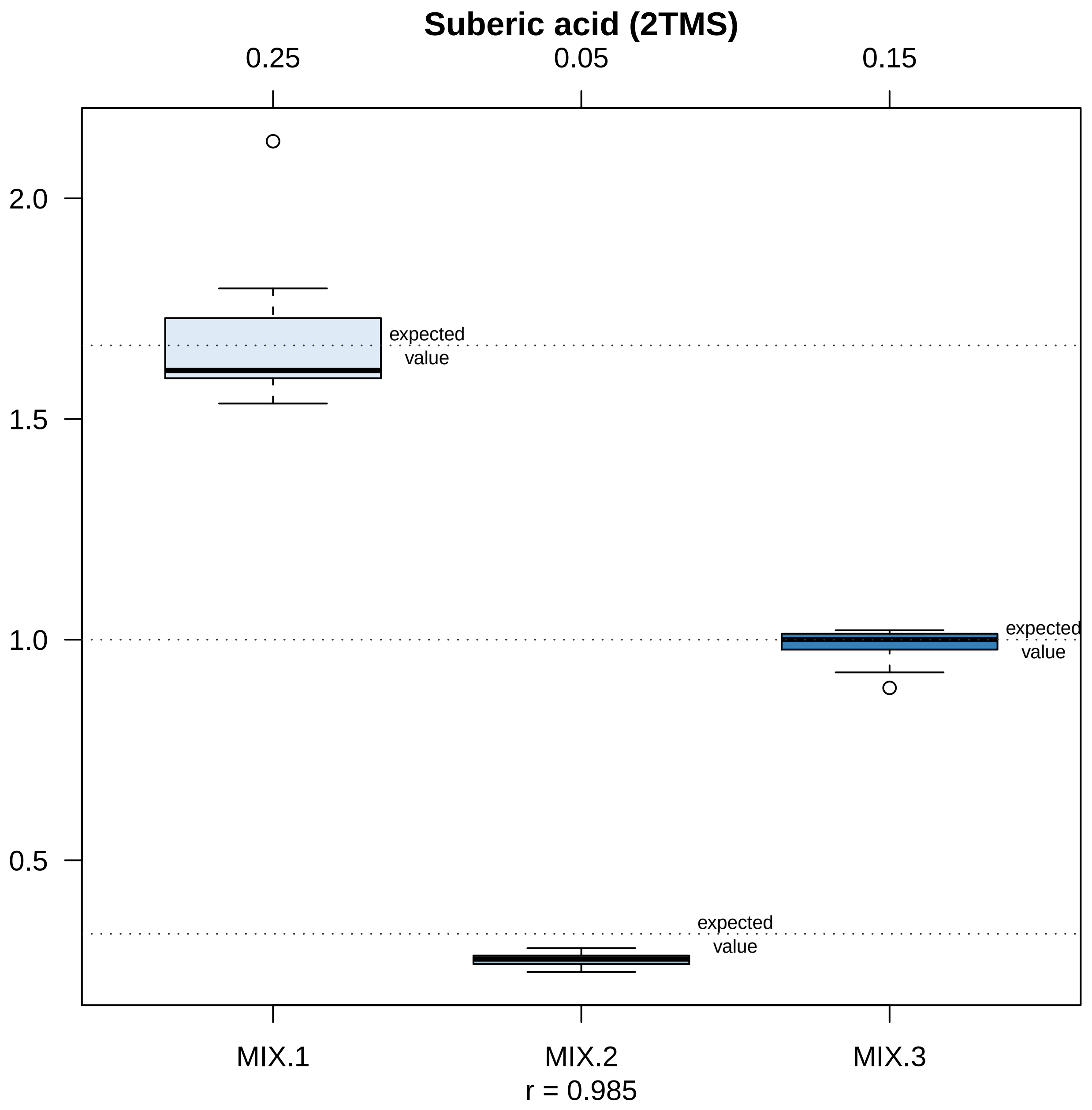

Suberic acid (2TMS)
0.25
0.05
0.15
2.0
1.5
1.0
0.5
expected value
expected value
expected value
MIX.1
MIX.2
MIX.3
r = 0.985

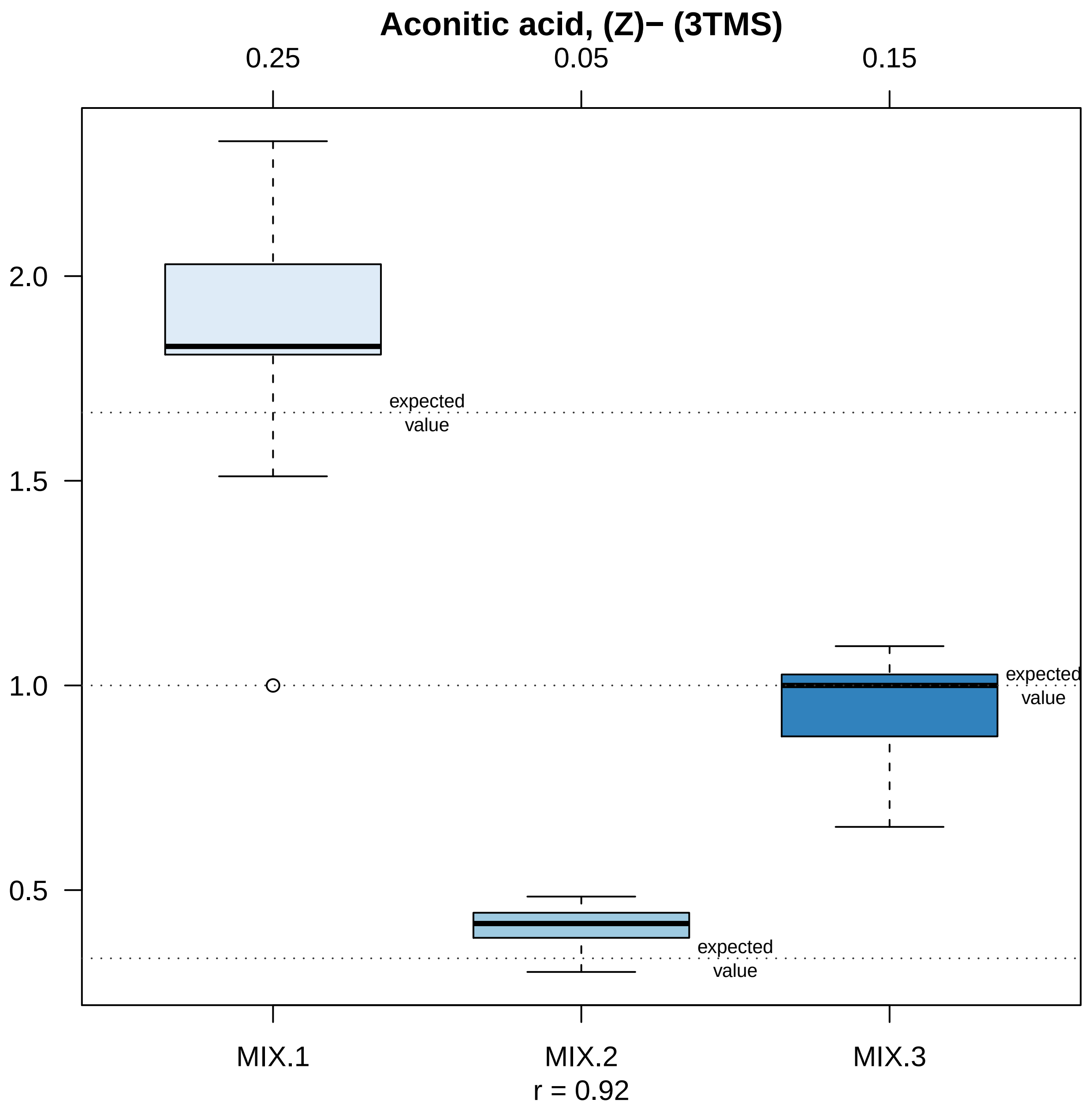

Aconitic acid, (Z)− (3TMS)
0.25
0.05
0.15
2.0
1.5
1.0
0.5
expected
value
expected
value
expected
value
MIX.1
MIX.2
MIX.3
r = 0.92

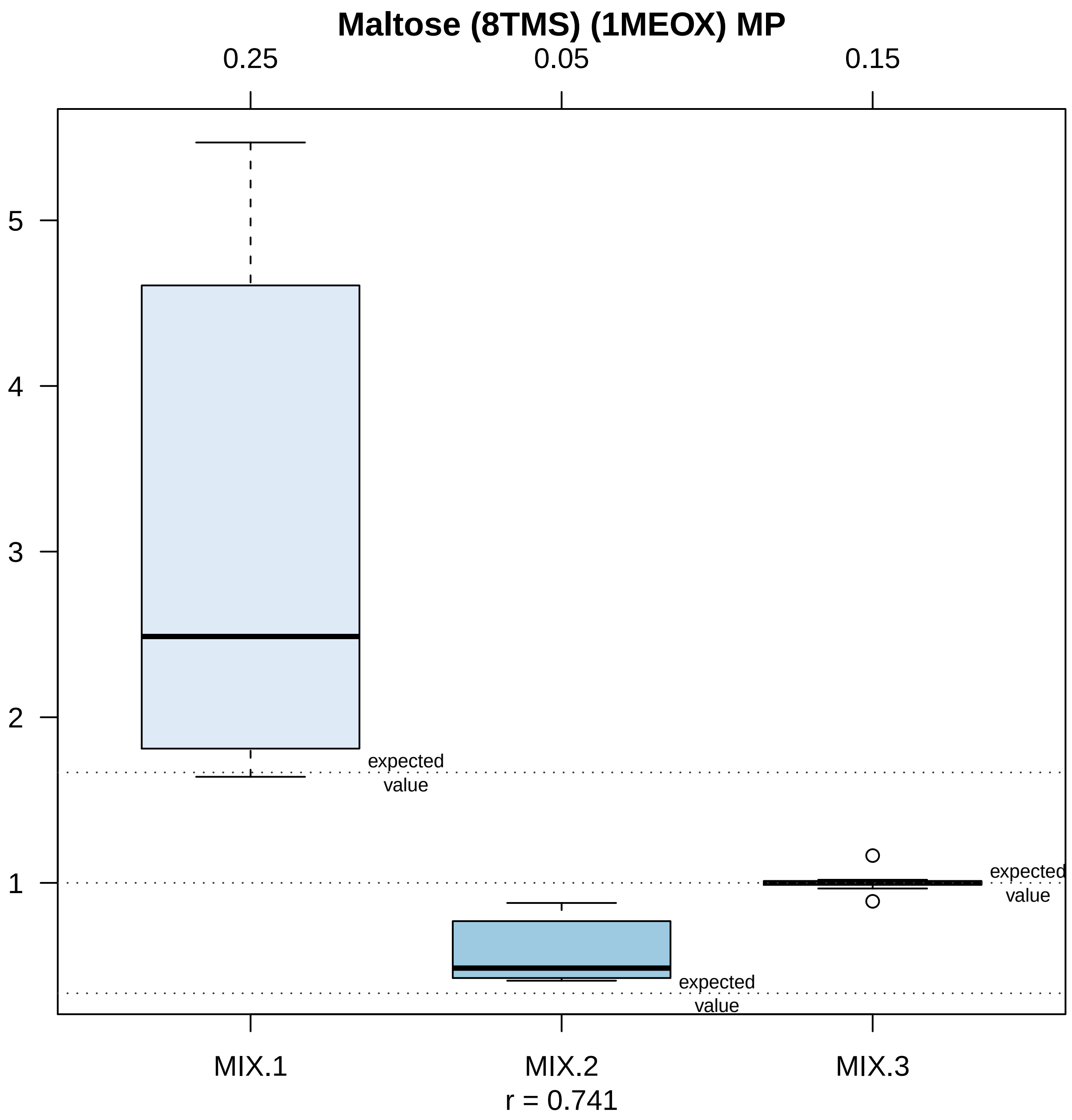

Maltose (8TMS) (1MEOX) MP
0.25
0.05
0.15
5
4
3
2
1
expected value
expected value
expected value
MIX.1
MIX.2
MIX.3
r = 0.741

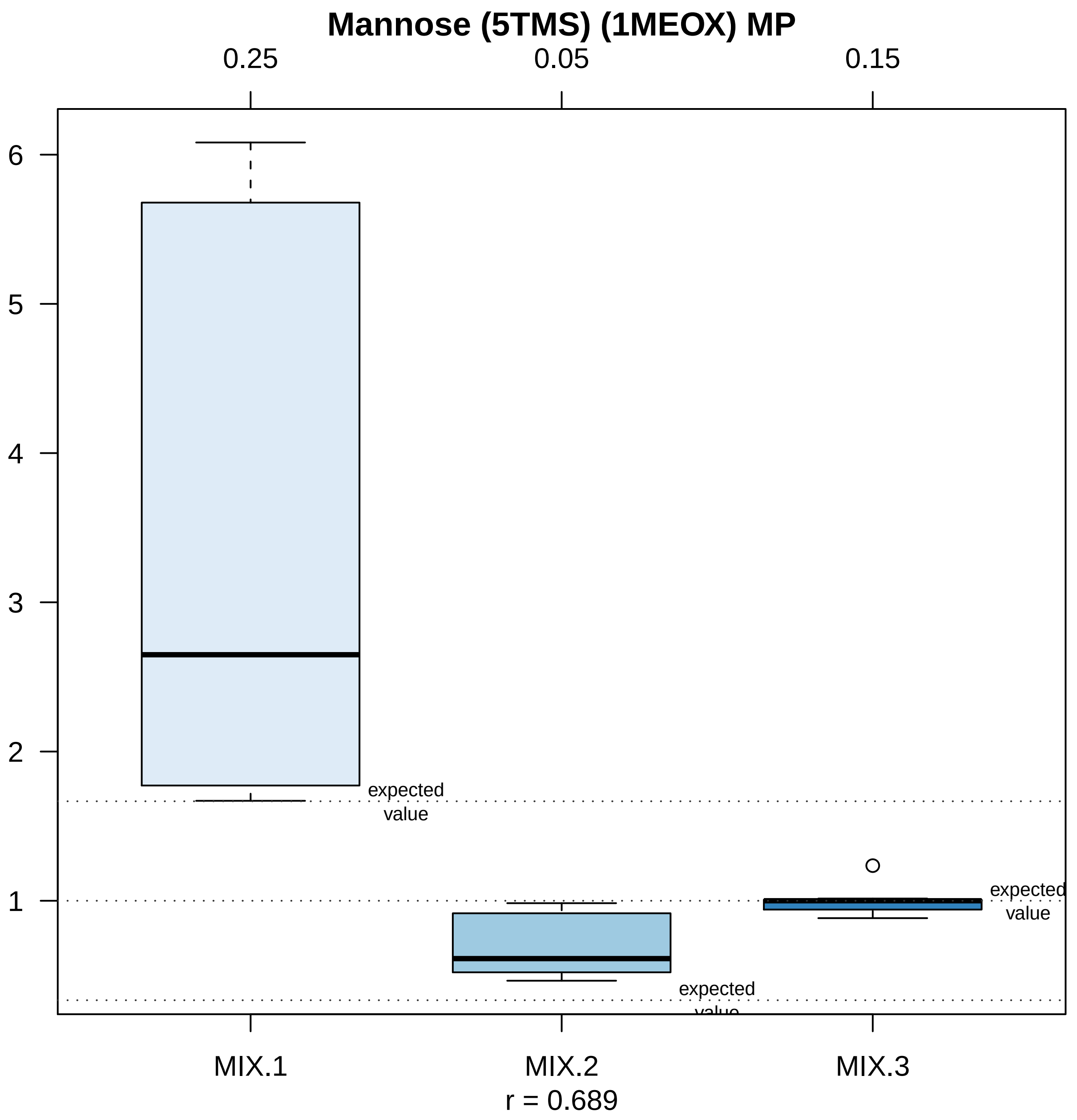
Mannose (5TMS) (1MEOX) MP
0.25
0.05
0.15
6
5
4
3
2
1
expected
value
expected
value
expected
value
MIX.1
MIX.2
MIX.3
r = 0.689

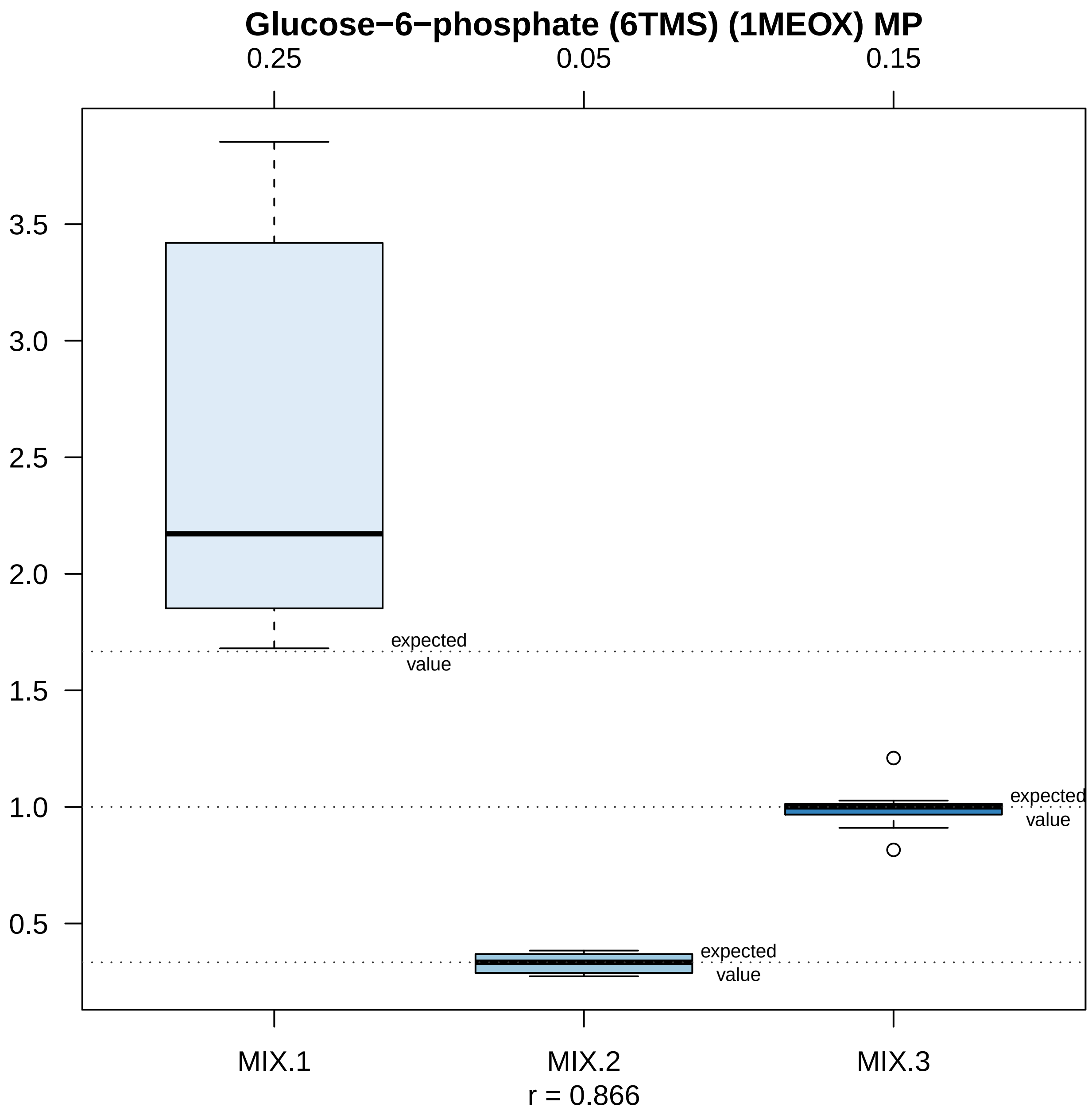

Glucose−6−phosphate (6TMS) (1MEOX) MP
0.25
0.05
0.15
3.5
3.0
2.5
2.0
1.5
1.0
0.5
expected value
expected value
expected value
MIX.1
MIX.2
MIX.3
r = 0.866

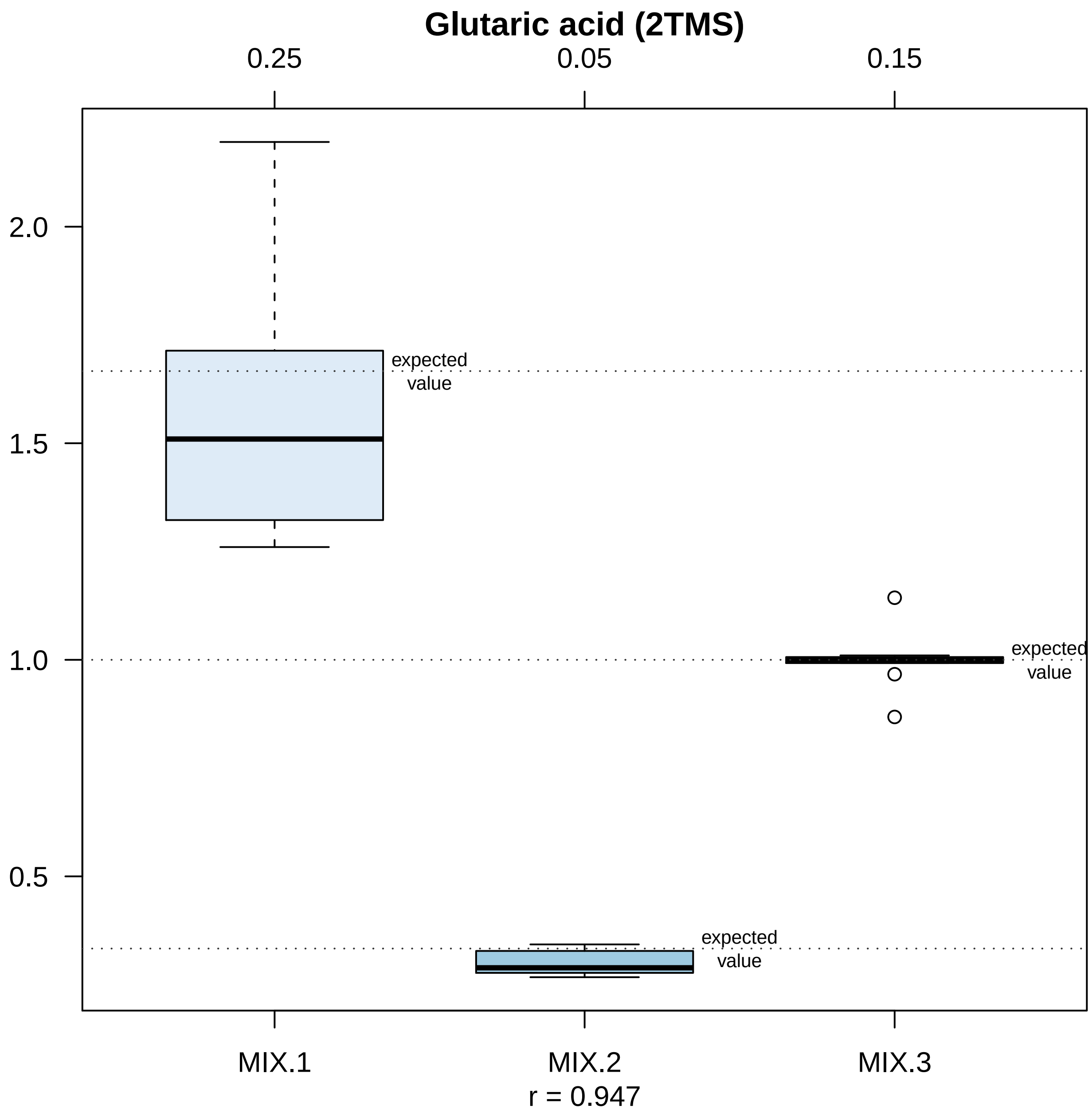
Glutaric acid (2TMS)
0.25
0.05
0.15
2.0
1.5
1.0
0.5
expected value
expected value
expected value
MIX.1
MIX.2
MIX.3
r = 0.947

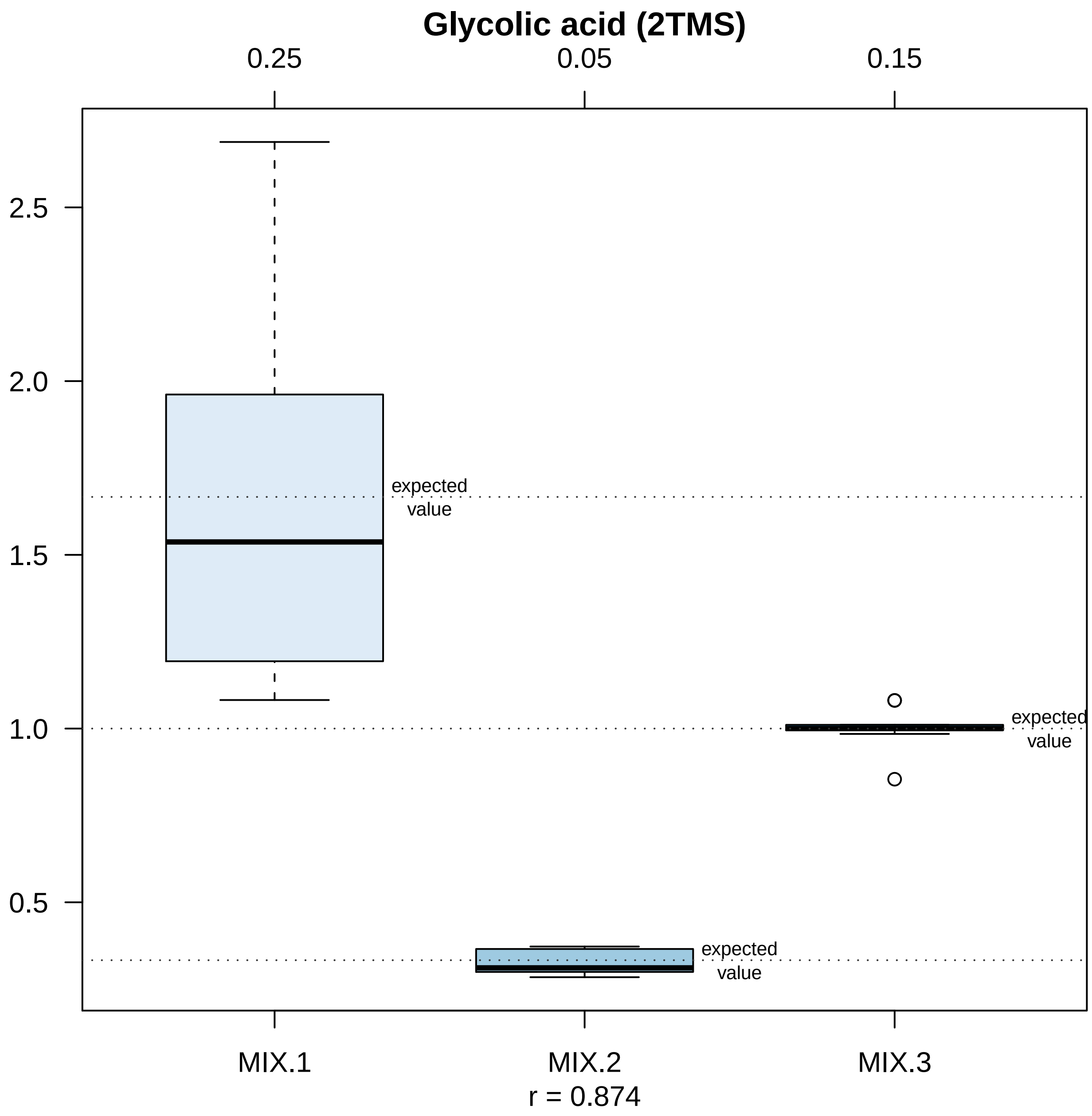

Glycolic acid (2TMS)
0.25
0.05
0.15
2.5
2.0
1.5
1.0
0.5
expected value
expected value
expected value
MIX.1
MIX.2
MIX.3
r = 0.874

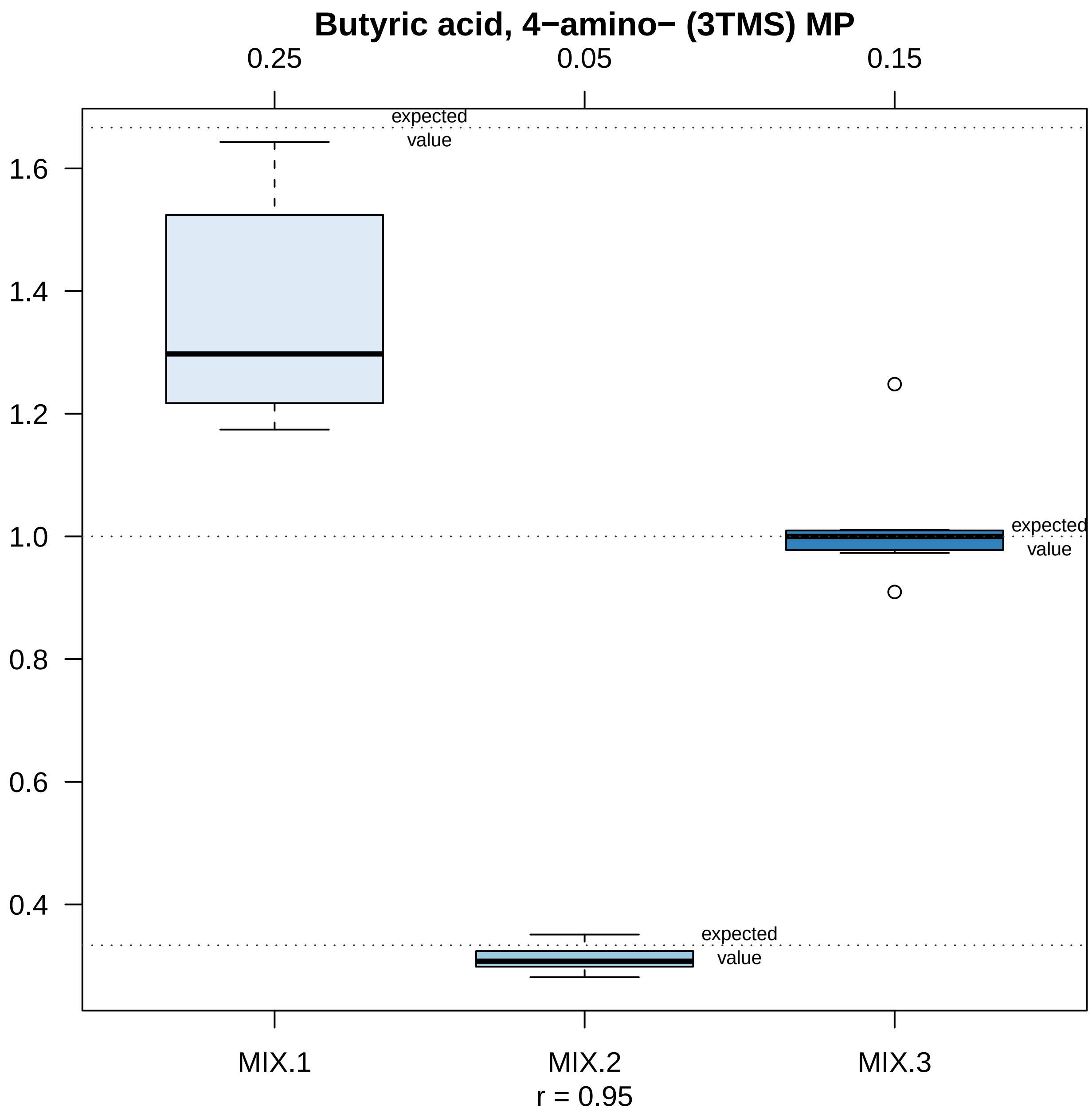

Butyric acid, 4−amino− (3TMS) MP
0.25
0.05
0.15
1.6
1.4
1.2
1.0
0.8
0.6
0.4
expected value
expected value
expected value
MIX.1
MIX.2
MIX.3
r = 0.95

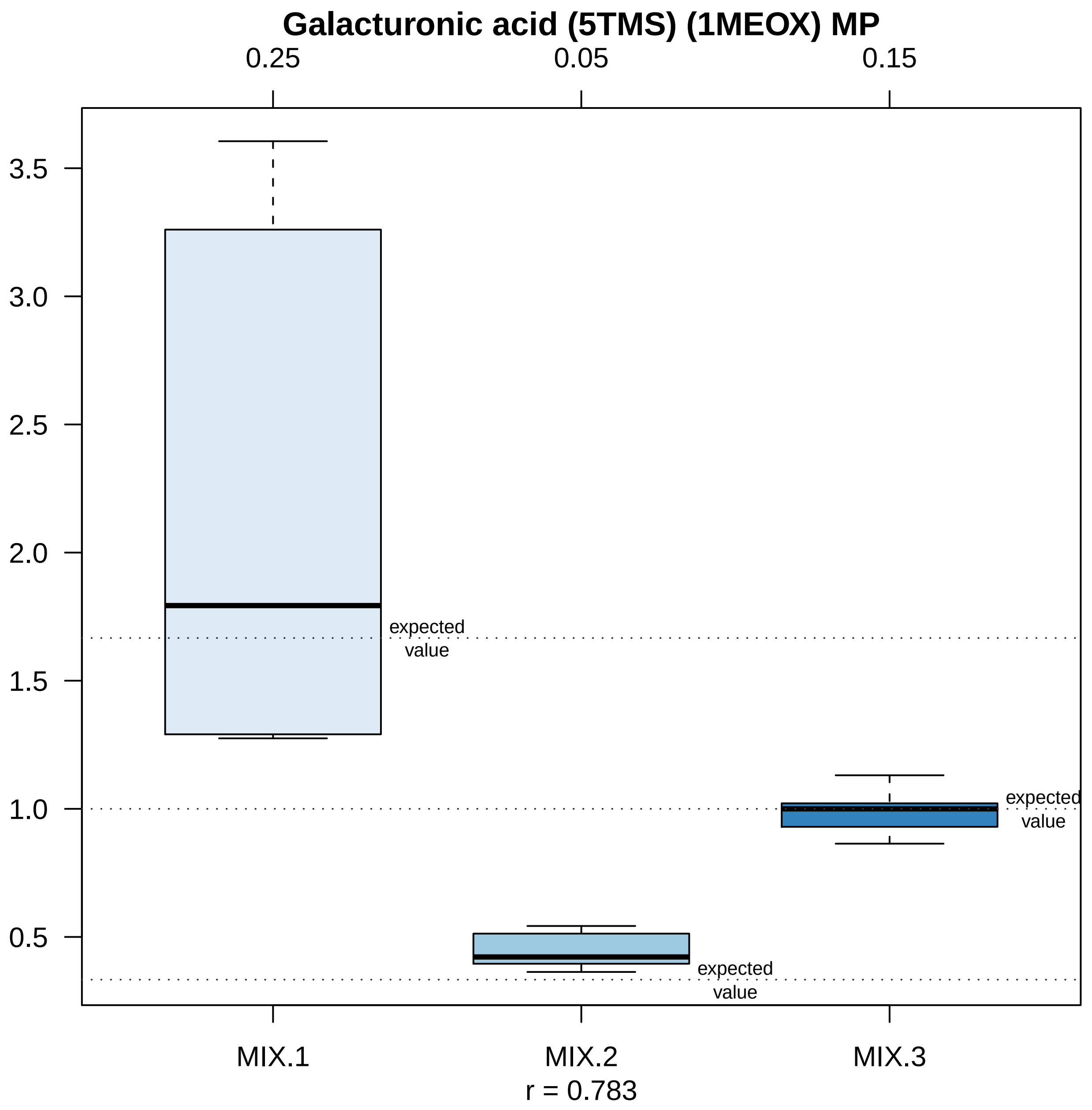
Galacturonic acid (5TMS) (1MEOX) MP
0.25
0.05
0.15
3.5
3.0
2.5
2.0
1.5
1.0
0.5
expected value
expected value
expected value
MIX.1
MIX.2
MIX.3
r = 0.783

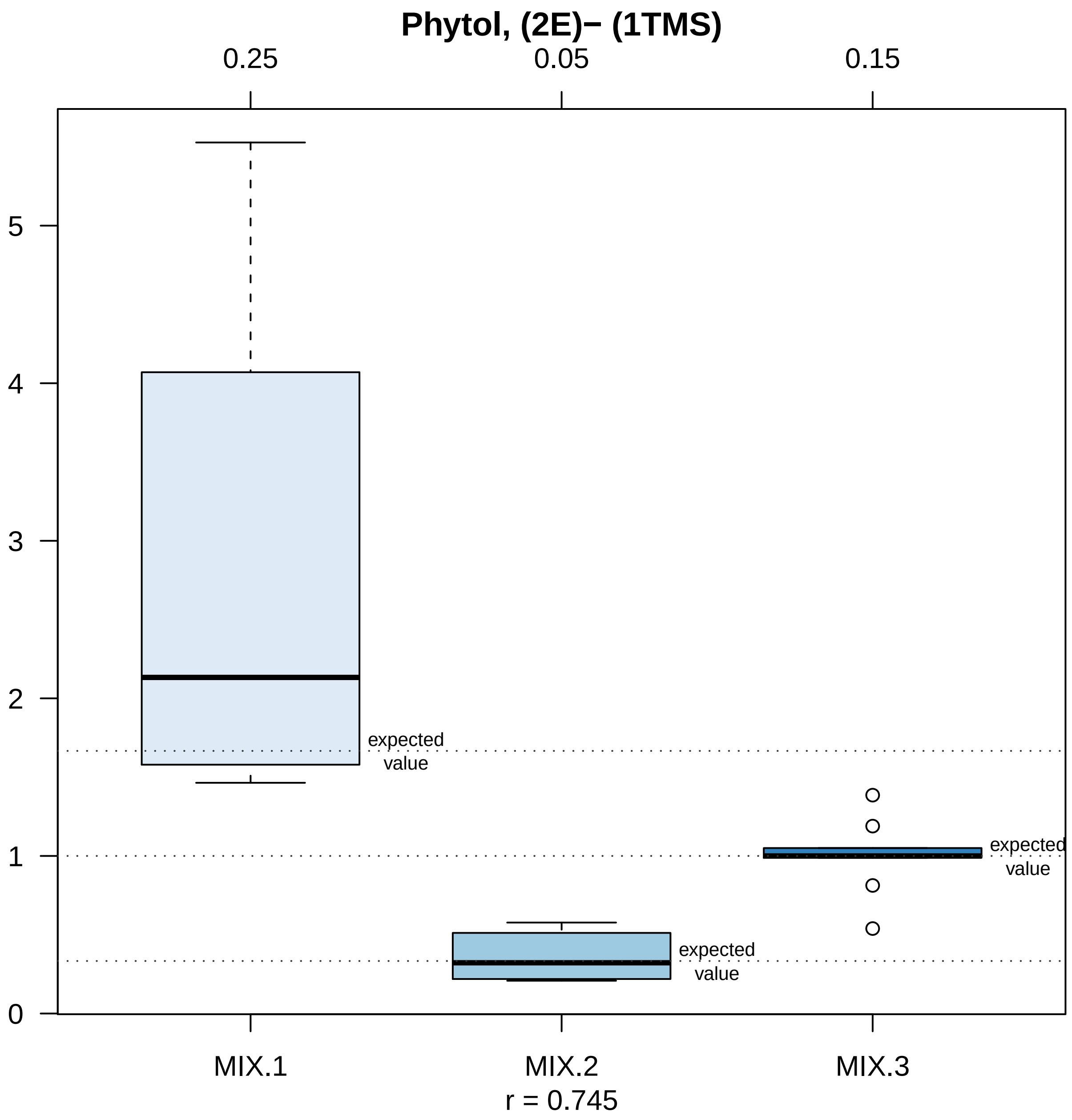

Phytol, (2E)− (1TMS)
0.25
0.05
0.15
5
4
3
2
1
0
expected value
expected value
expected value
MIX.1
MIX.2
MIX.3
r = 0.745

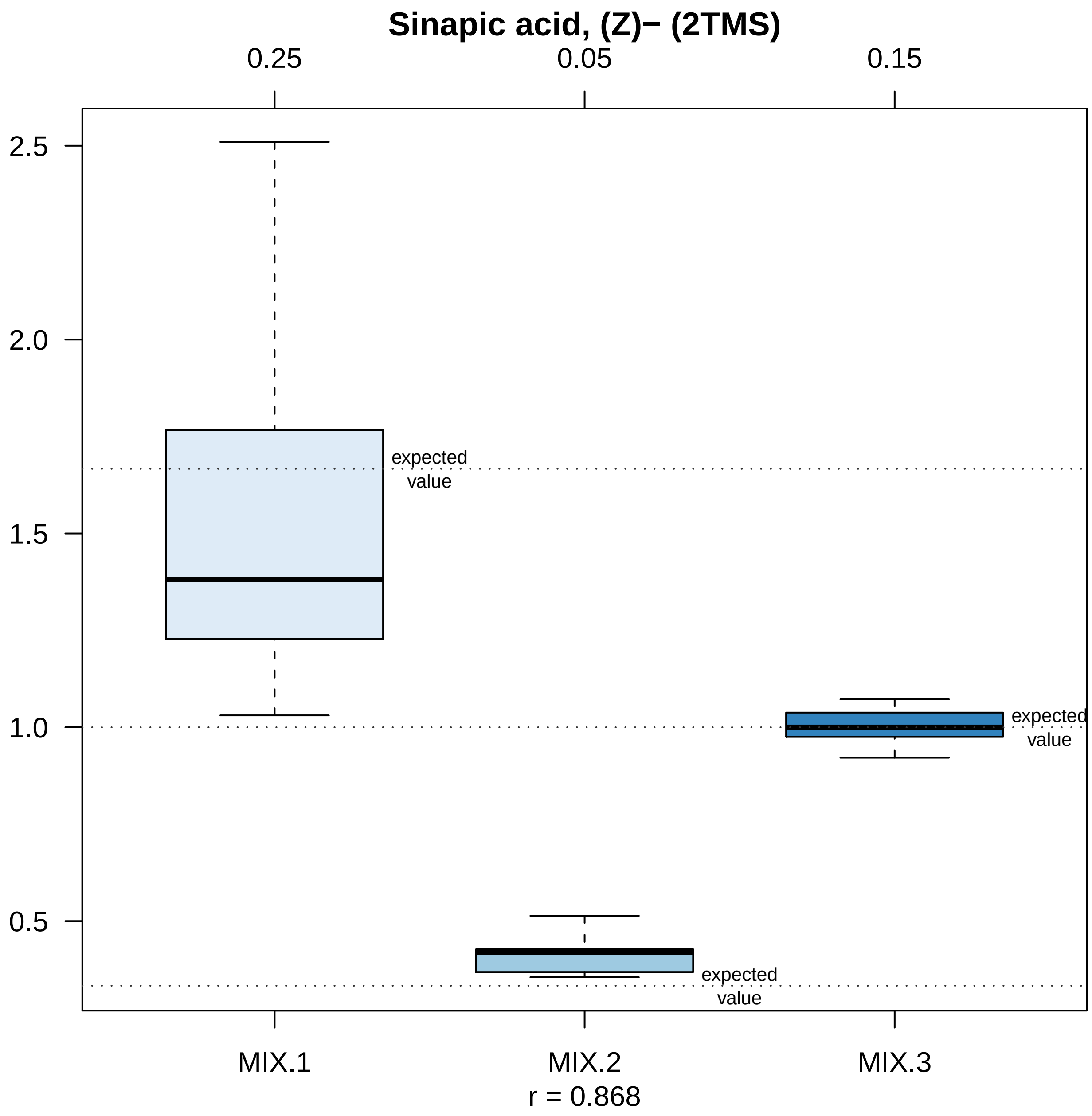
Sinapic acid, (Z)− (2TMS)
0.25
0.05
0.15
2.5
2.0
1.5
1.0
0.5
expected value
expected value
expected value
MIX.1
MIX.2
MIX.3
r = 0.868

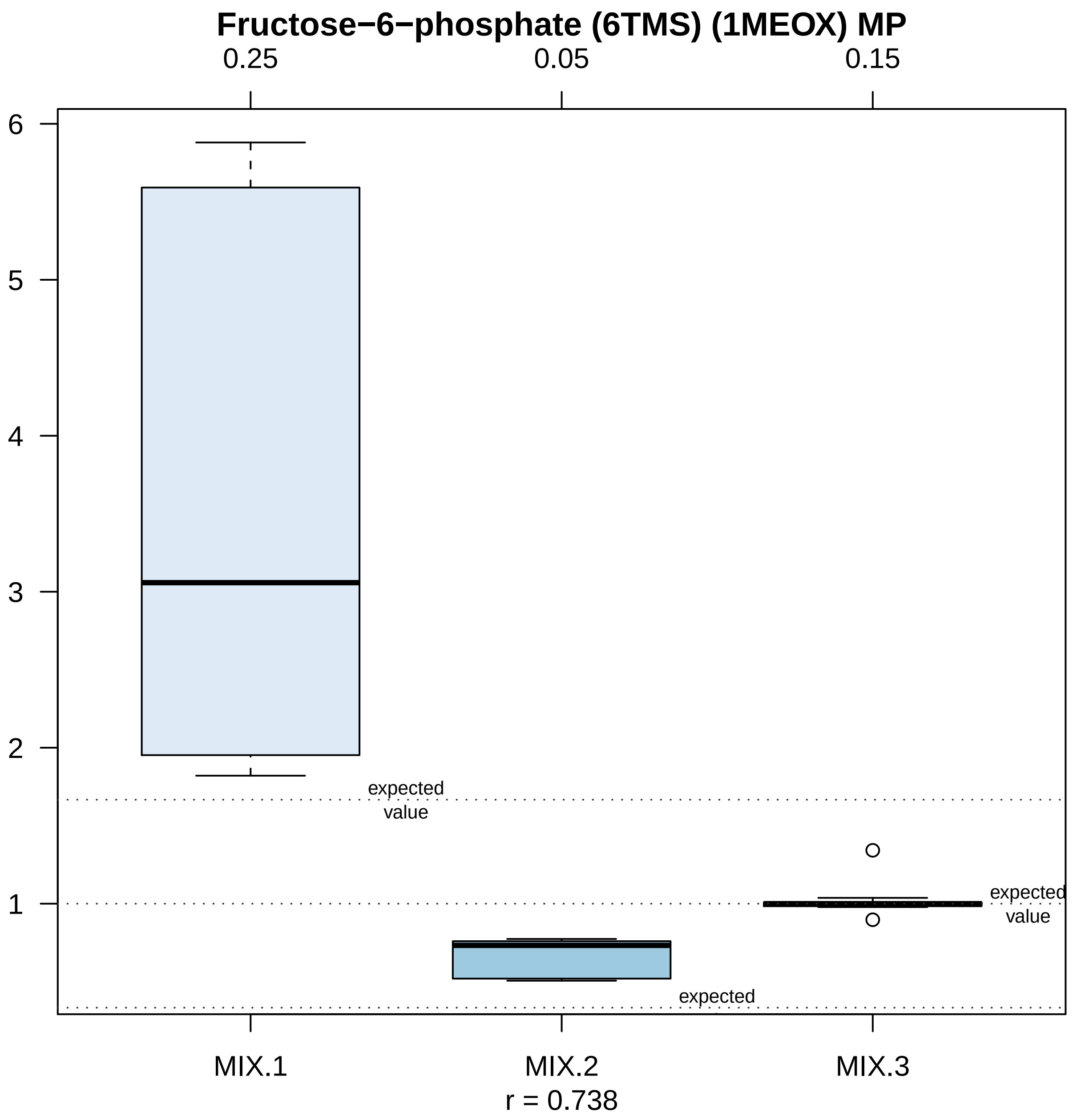
Fructose−6−phosphate (6TMS) (1MEOX) MP
0.25
0.05
0.15
6
5
4
3
2
1
expected
value
expected
value
expected
MIX.1
MIX.2
MIX.3
r = 0.738

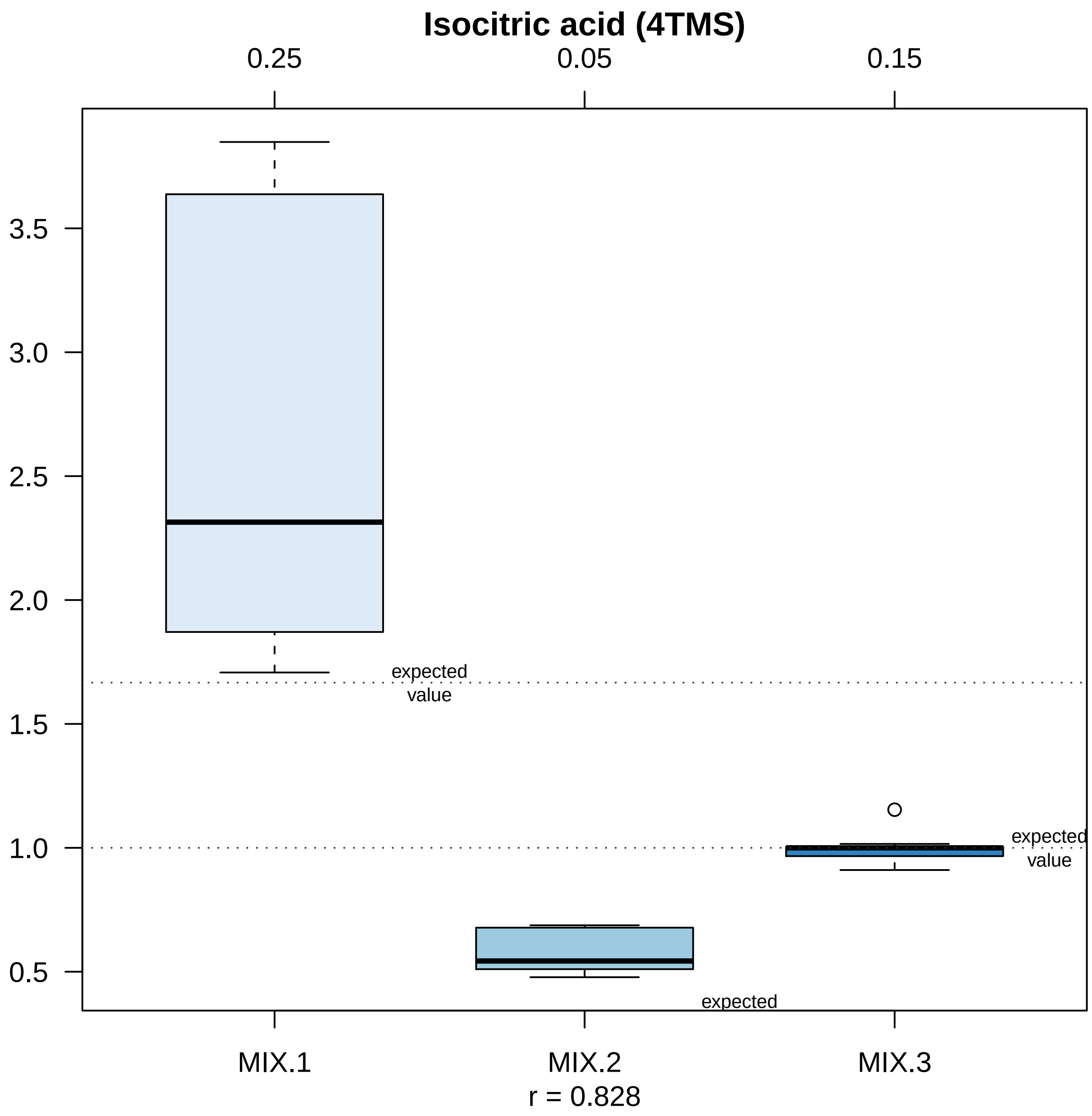

Isocitric acid (4TMS)
0.25
0.05
0.15
3.5
3.0
2.5
2.0
1.5
1.0
0.5
expected
value
expected
value
expected
MIX.1
MIX.2
MIX.3
r = 0.828

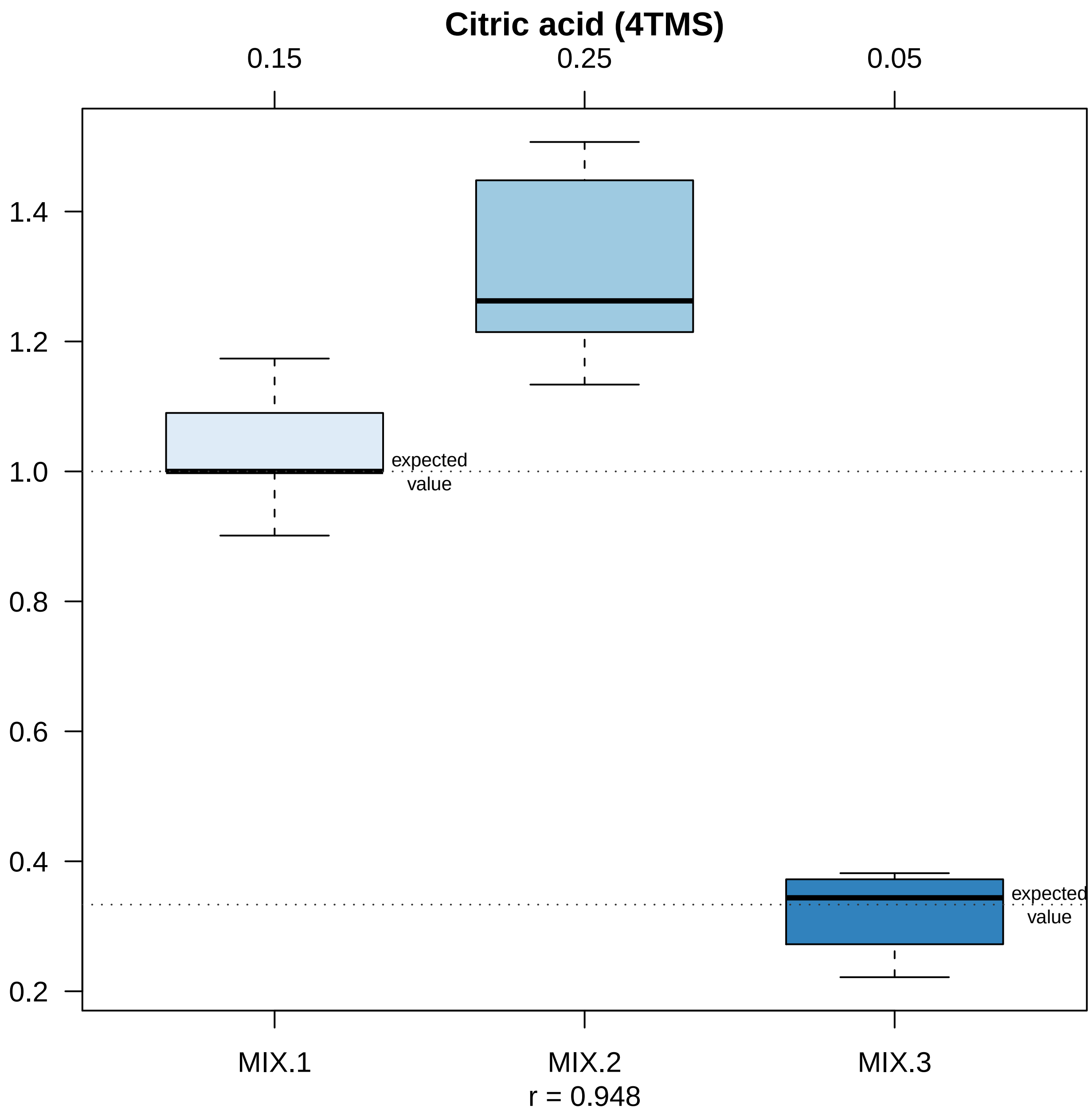
Citric acid (4TMS)
0.15
0.25
0.05
1.4
1.2
1.0
0.8
0.6
0.4
0.2
expected value
expected value
MIX.1
MIX.2
MIX.3
r = 0.948

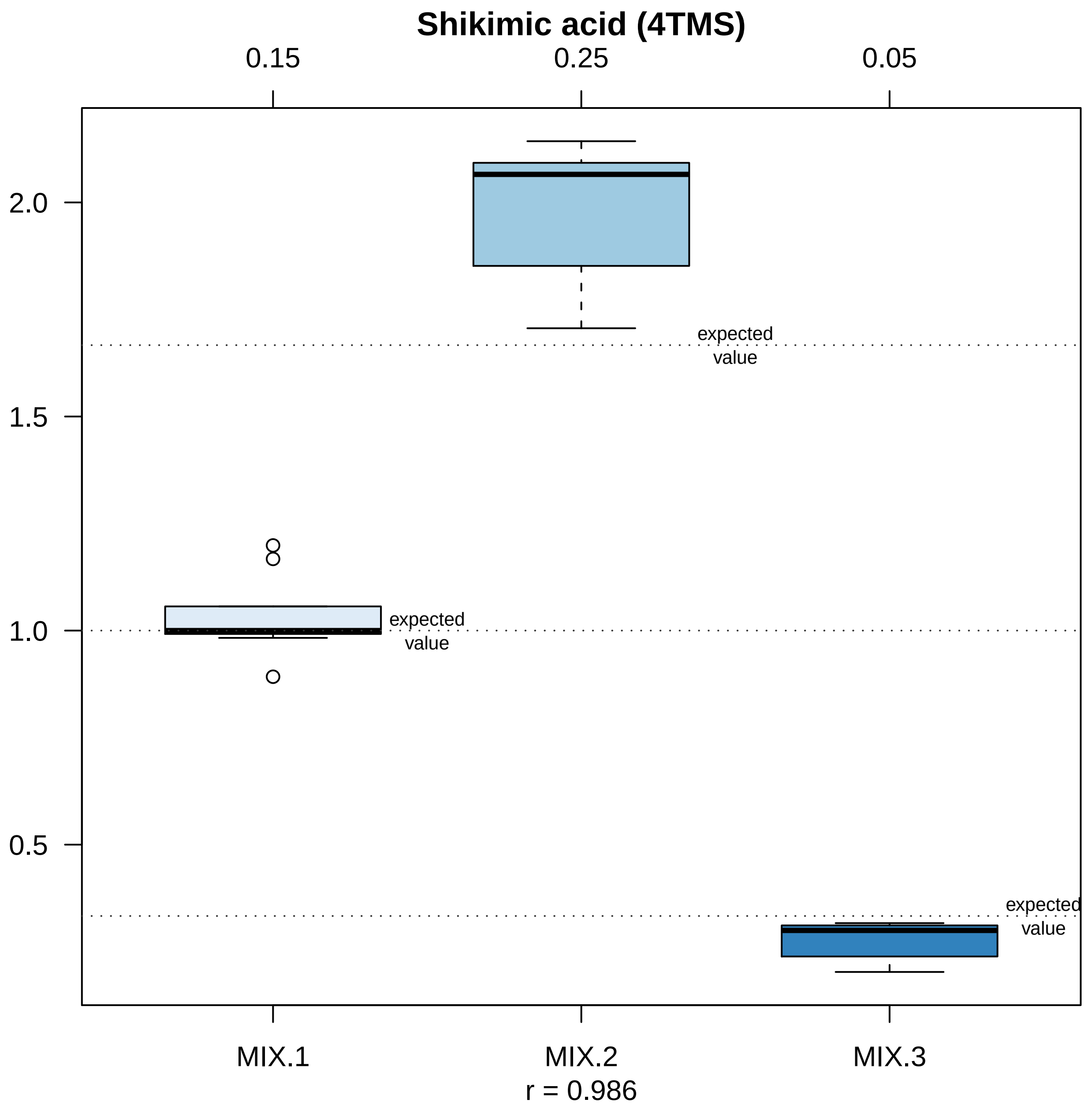

Shikimic acid (4TMS)
0.15
0.25
0.05
2.0
1.5
1.0
0.5
expected value
expected value
expected value
MIX.1
MIX.2
MIX.3
r = 0.986

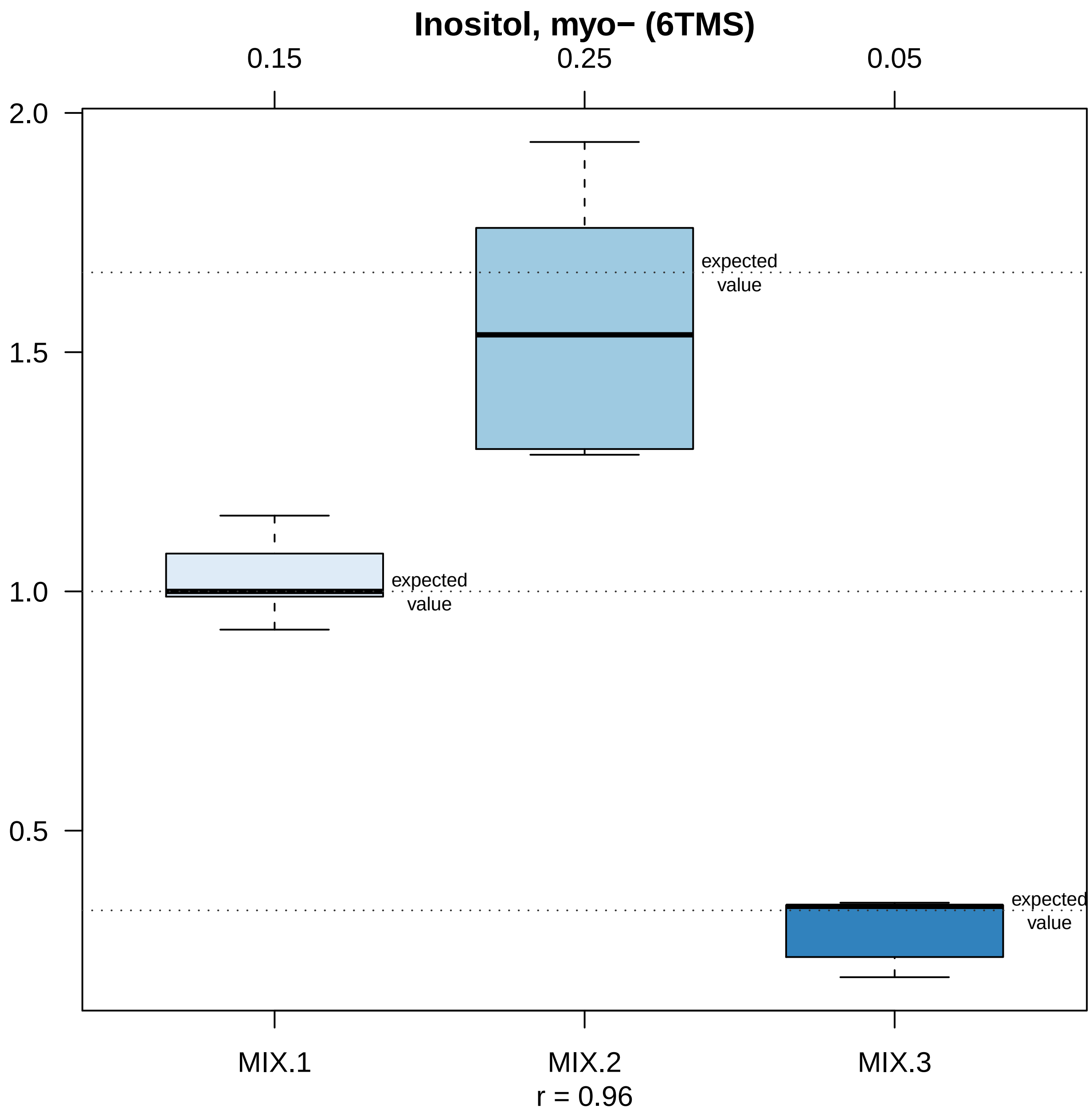

Inositol, myo− (6TMS)
0.15
0.25
0.05
2.0
1.5
1.0
0.5
expected
value
expected
value
expected
value
MIX.1
MIX.2
MIX.3
r = 0.96

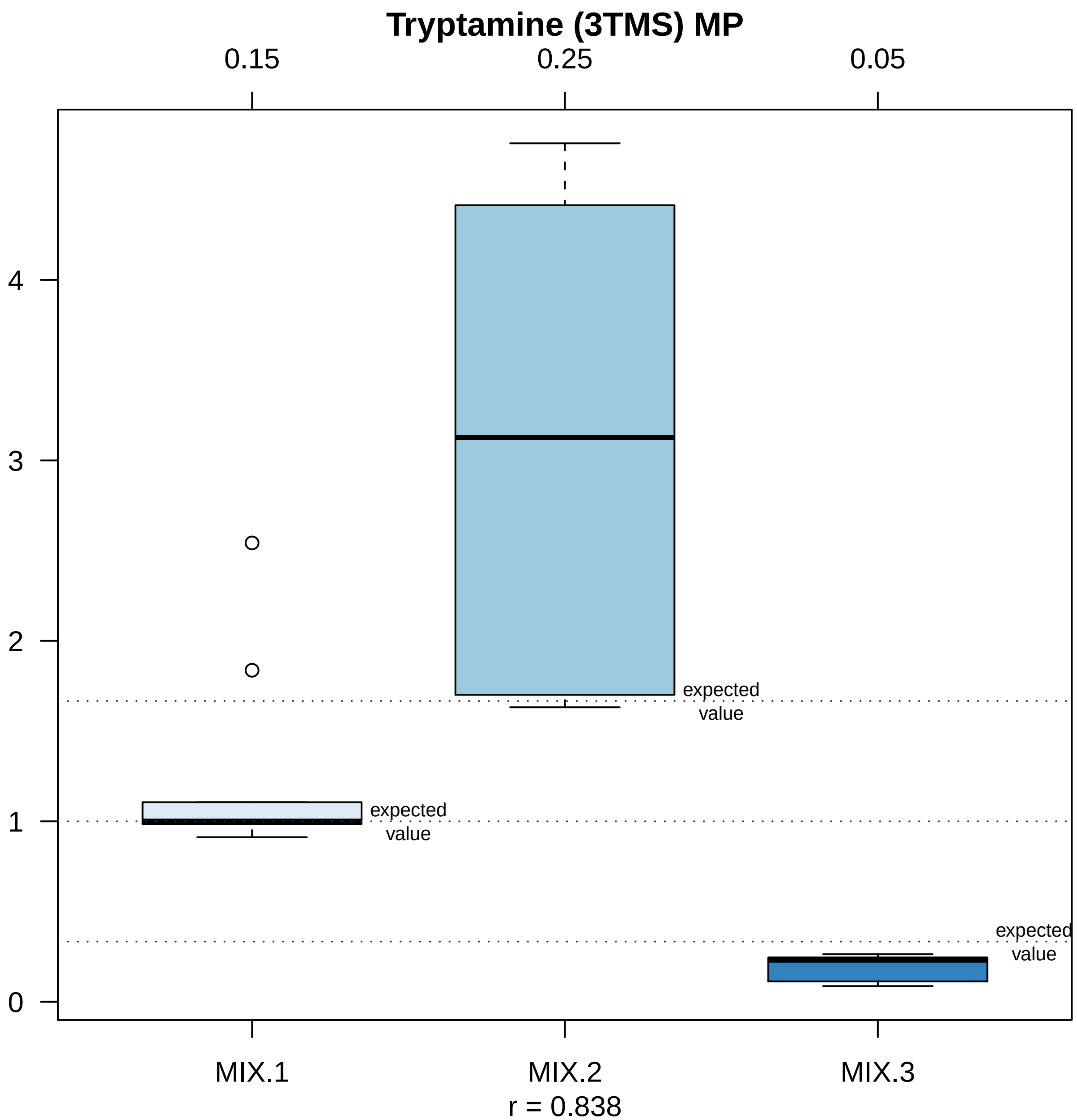
Tryptamine (3TMS) MP
0.15
0.25
0.05
4
3
2
1
0
expected value
expected value
expected value
MIX.1
MIX.2
MIX.3
r = 0.838

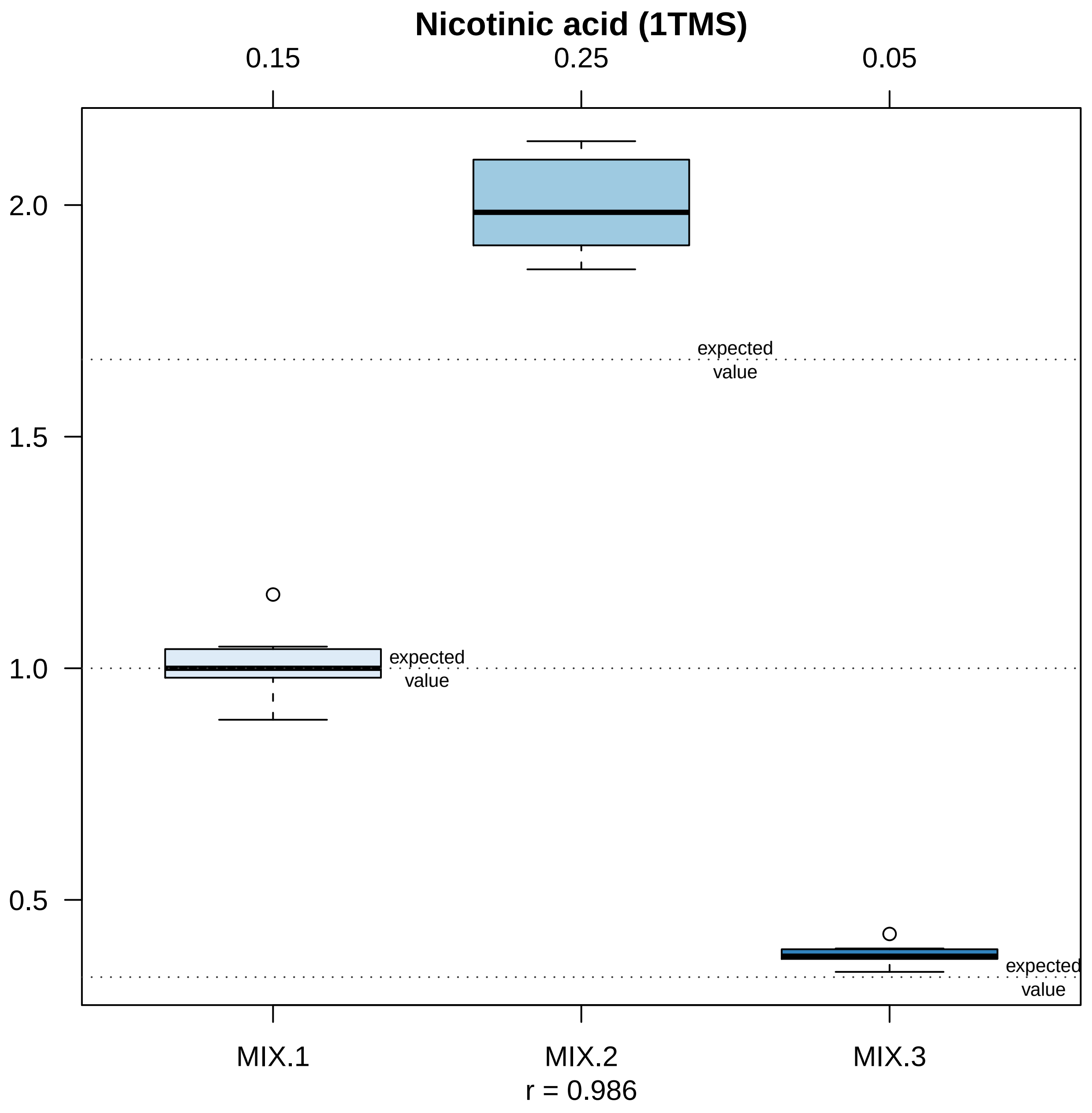

Nicotinic acid (1TMS)
0.15
0.25
0.05
2.0
1.5
1.0
0.5
expected value
expected value
expected value
MIX.1
MIX.2
MIX.3
r = 0.986

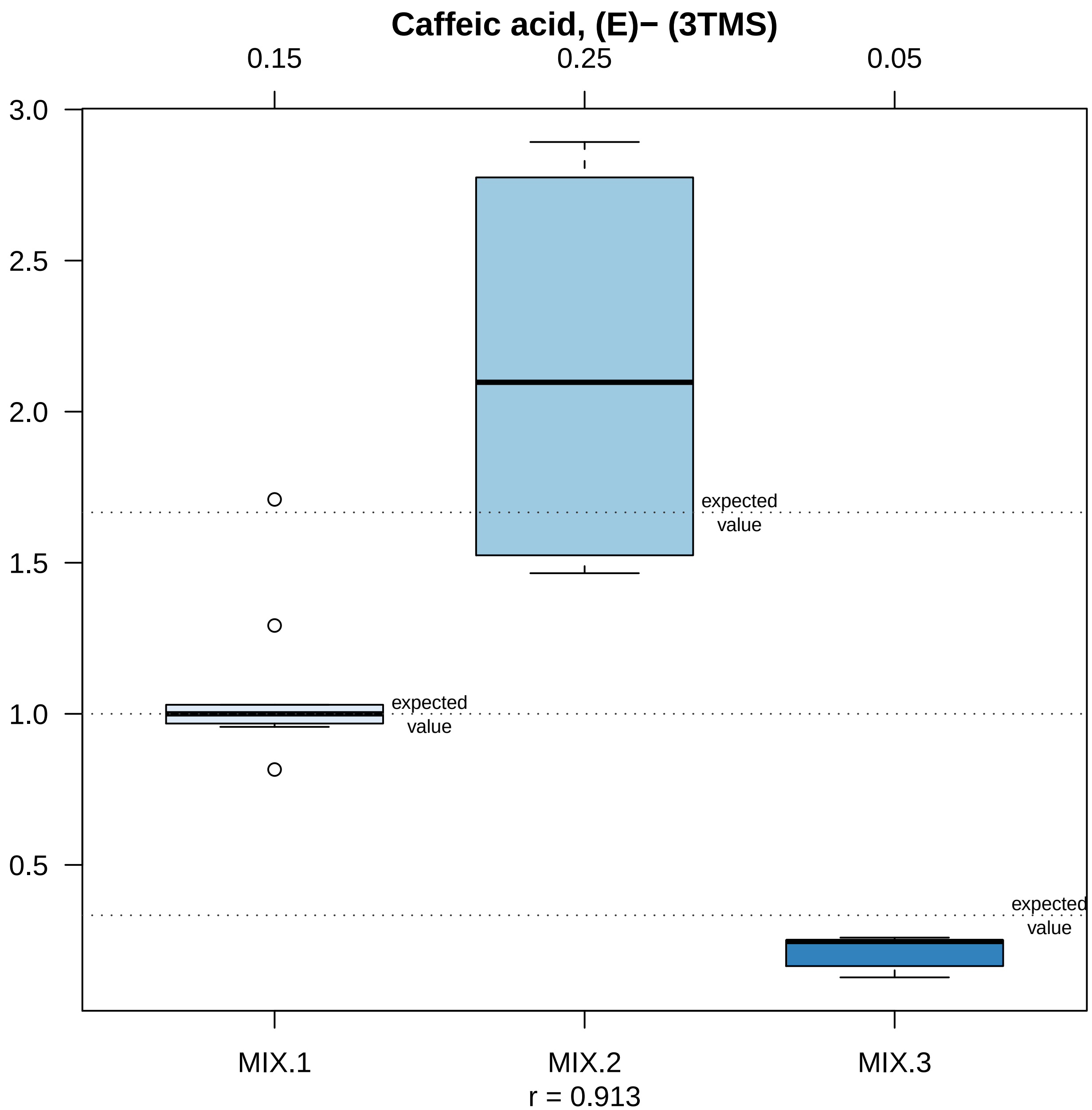
Caffeic acid, (E)− (3TMS)
0.15
0.25
0.05
3.0
2.5
2.0
1.5
1.0
0.5
expected value
expected value
expected value
MIX.1
MIX.2
MIX.3
r = 0.913

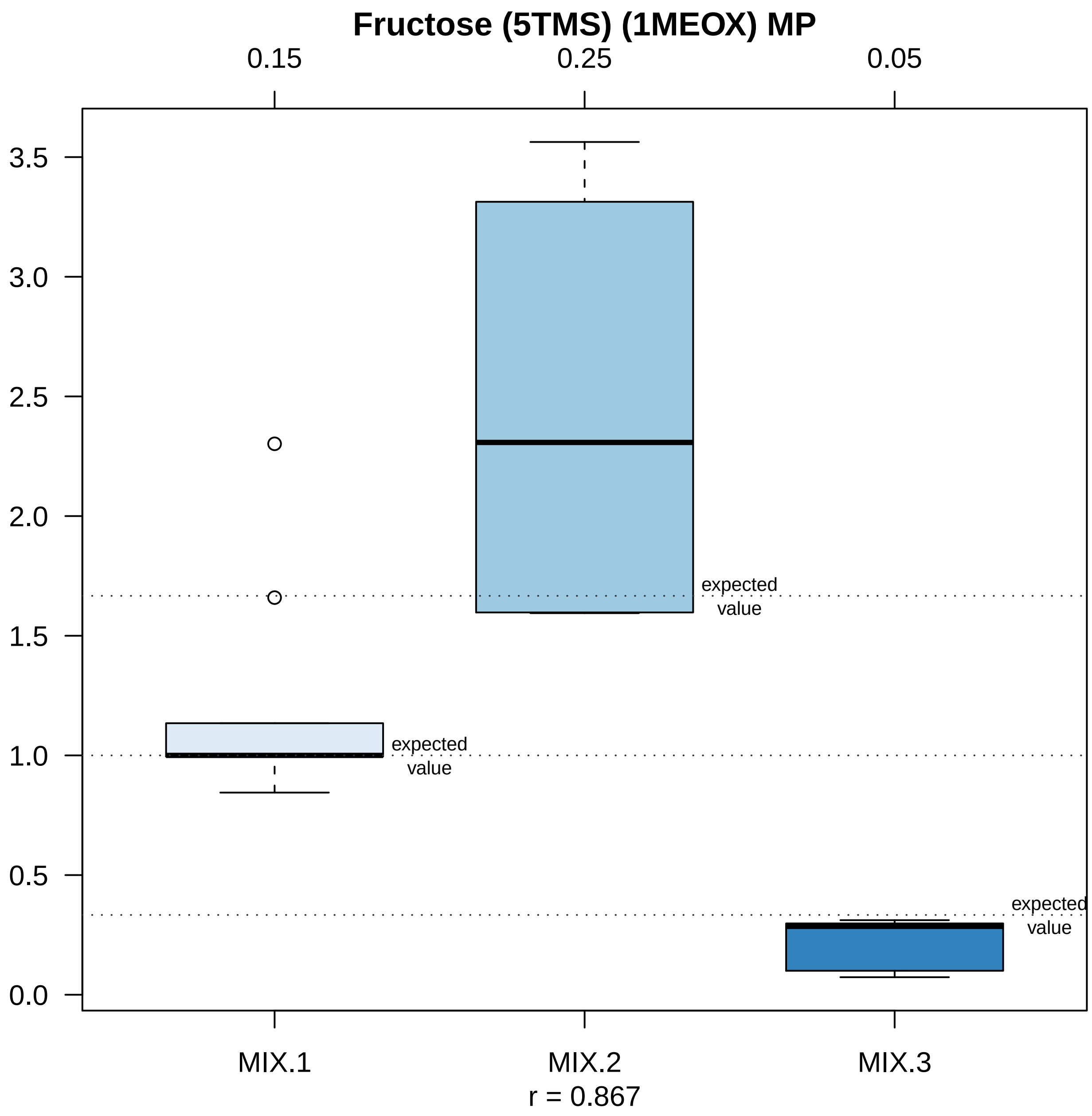

Fructose (5TMS) (1MEOX) MP
0.15
0.25
0.05
3.5
3.0
2.5
2.0
1.5
1.0
0.5
0.0
expected value
expected value
expected value
MIX.1
MIX.2
MIX.3
r = 0.867

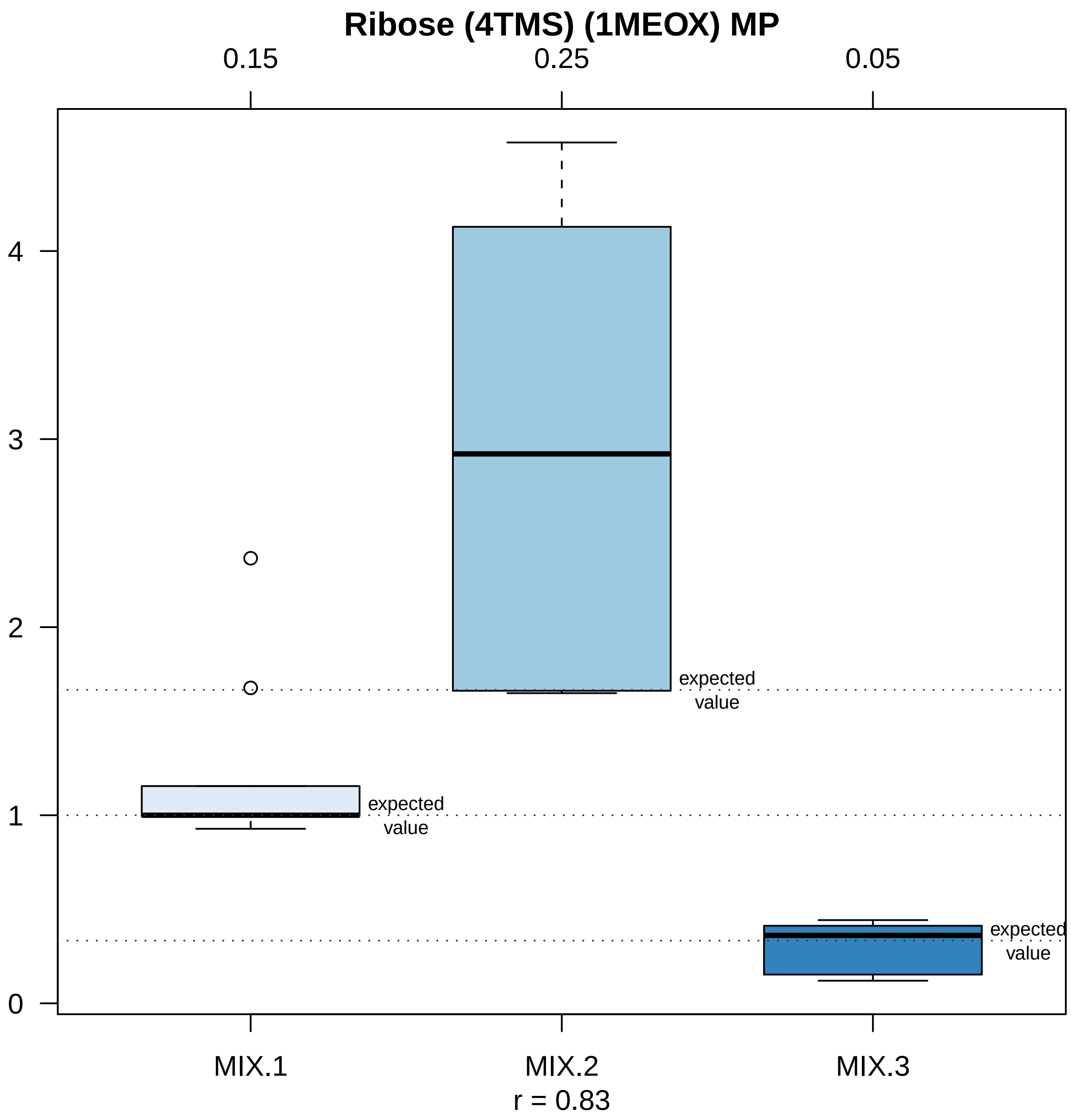
Ribose (4TMS) (1MEOX) MP
0.15
0.25
0.05
4
3
2
1
0
expected value
expected value
expected value
MIX.1
MIX.2
MIX.3
r = 0.83

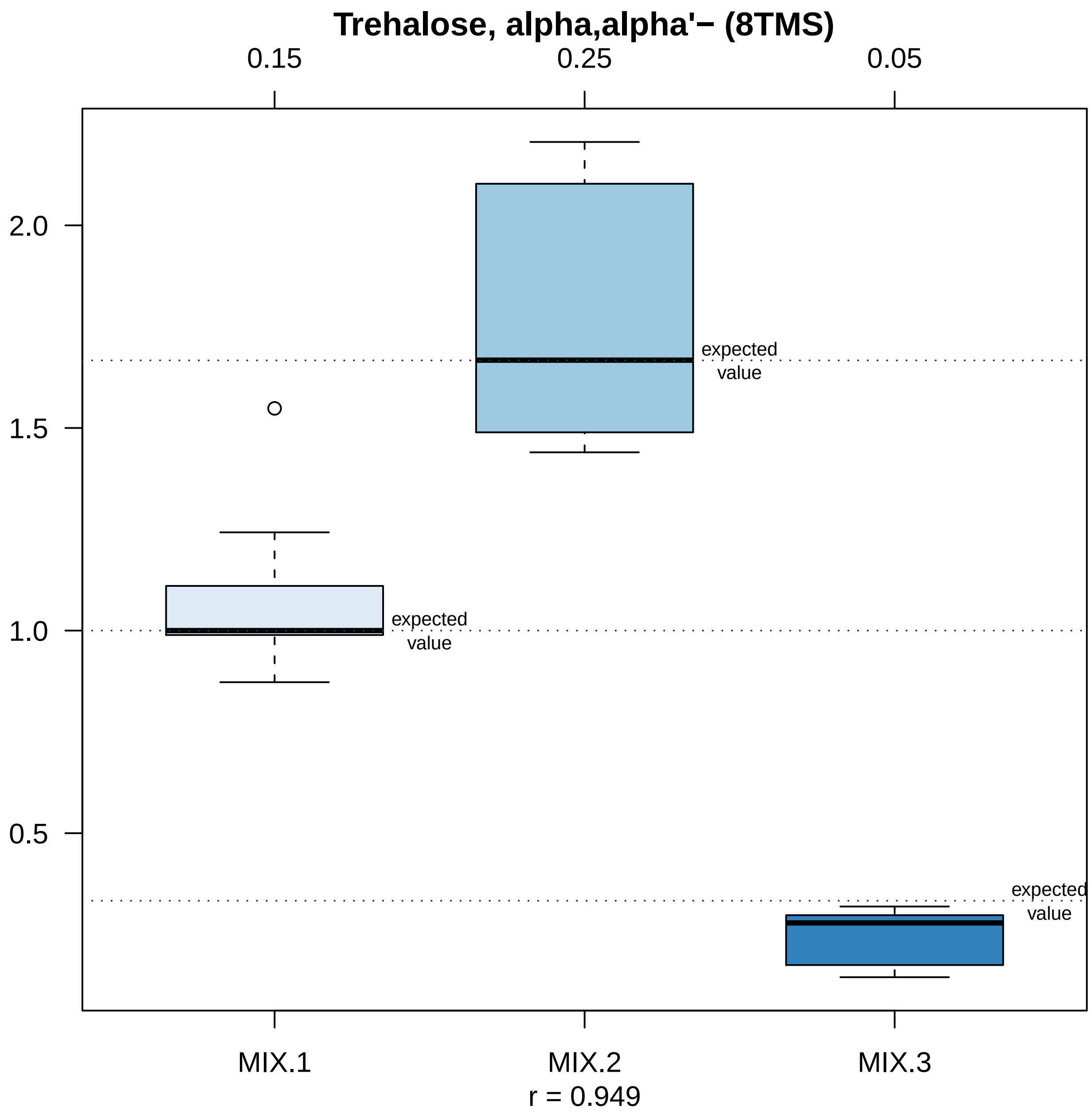

Trehalose, alpha,alpha'− (8TMS)
0.15
0.25
0.05
2.0
1.5
1.0
0.5
expected value
expected value
expected value
MIX.1
MIX.2
MIX.3
r = 0.949

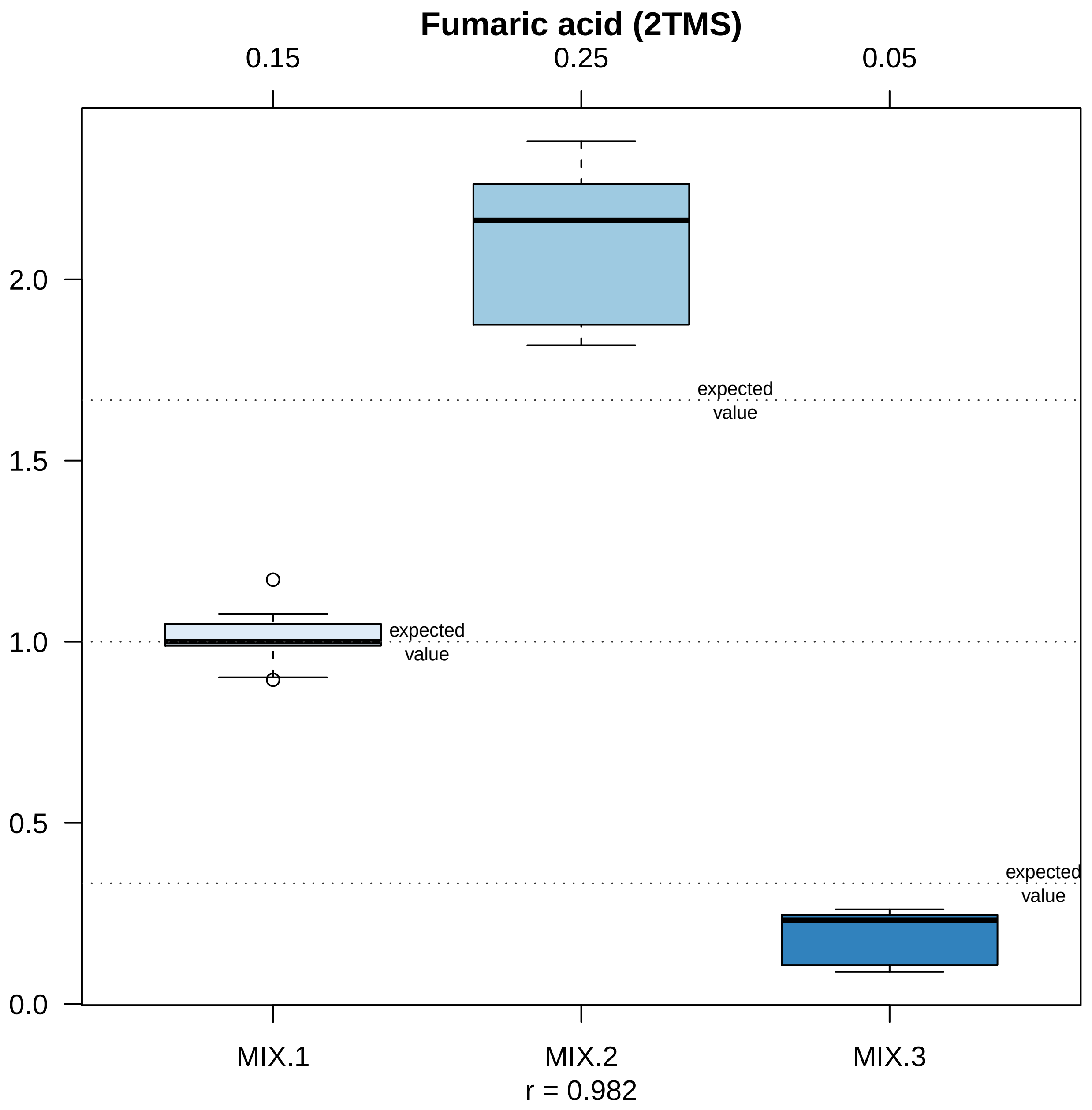
Fumaric acid (2TMS)
0.15
0.25
0.05
2.0
1.5
1.0
0.5
0.0
expected value
expected value
expected value
MIX.1
MIX.2
MIX.3
r = 0.982

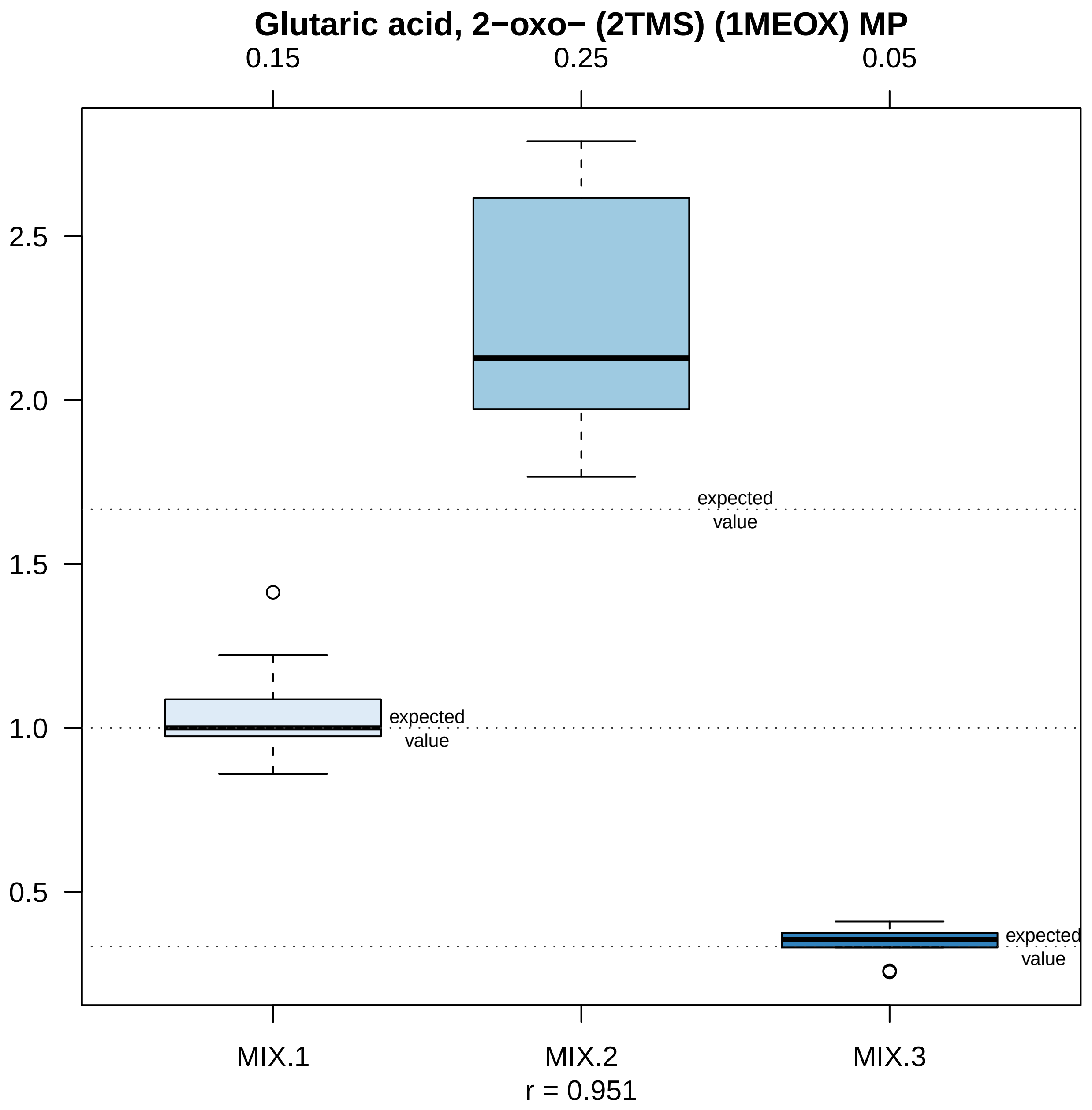
Glutaric acid, 2−oxo− (2TMS) (1MEOX) MP
0.15
0.25
0.05
2.5
2.0
1.5
1.0
0.5
expected value
expected value
expected value
MIX.1
MIX.2
MIX.3
r = 0.951

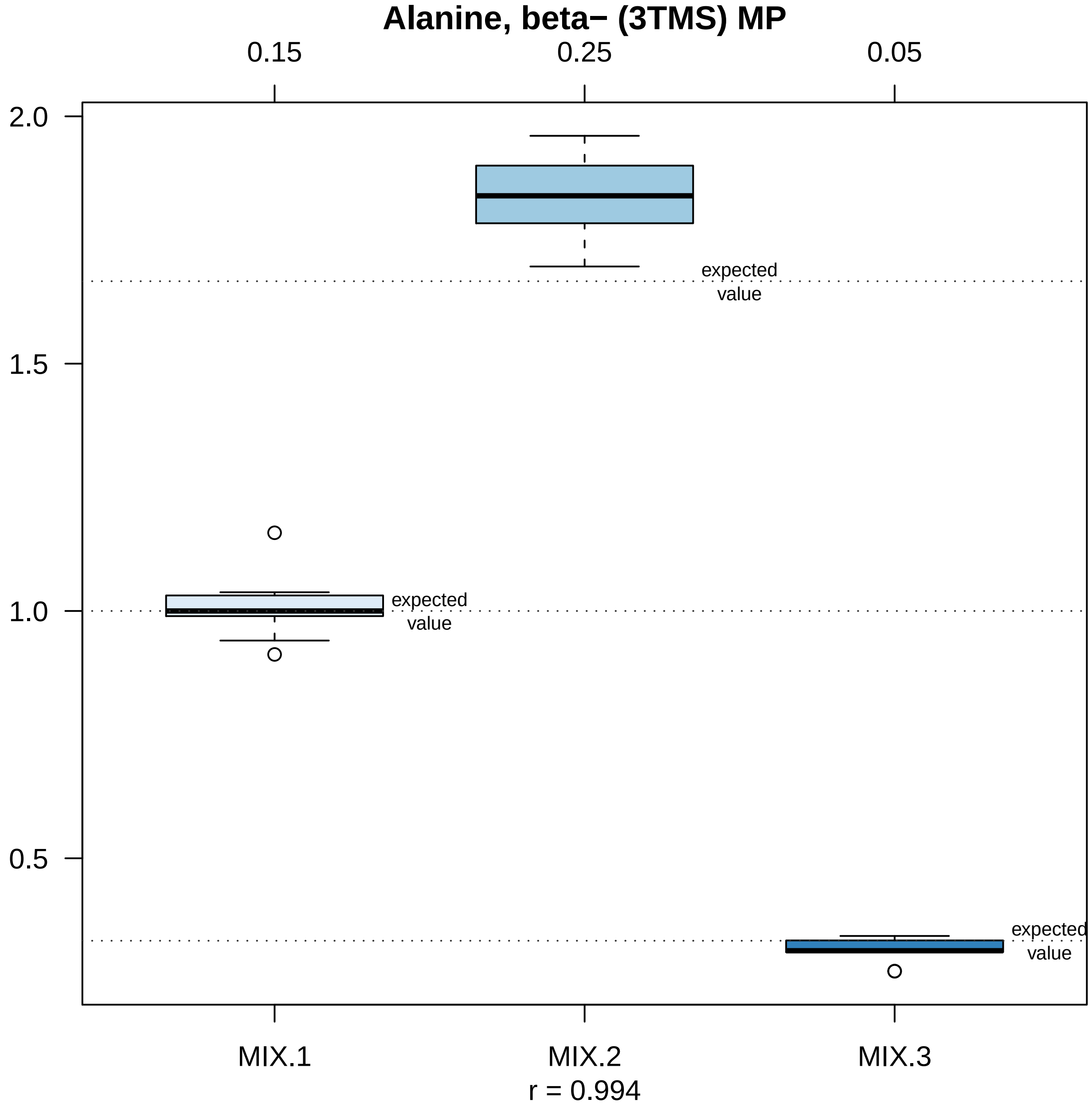

Alanine, beta− (3TMS) MP
0.15
0.25
0.05
2.0
1.5
1.0
0.5
expected value
expected value
expected value
MIX.1
MIX.2
MIX.3
r = 0.994
